# The ethical, social, and cultural dimensions of screening for mental health in children and adolescents of the developing world

**DOI:** 10.1371/journal.pone.0237853

**Published:** 2020-08-24

**Authors:** Fabio Salamanca-Buentello, Mary V. Seeman, Abdallah S. Daar, Ross E. G. Upshur

**Affiliations:** 1 Institute of Medical Science, University of Toronto, Toronto, Ontario, Canada; 2 Department of Psychiatry, University of Toronto, Toronto, Ontario, Canada; 3 Departments of Clinical Public Health and Surgery, University of Toronto, Toronto, Ontario, Canada; 4 Stellenbosch Institute for Advanced Study, Stellenbosch, Western Cape, South Africa; 5 Division of Clinical Public Health, Dalla Lana School of Public Health, University of Toronto, Toronto, Ontario, Canada; 6 Department of Family and Community Medicine, University of Toronto, Toronto, Ontario, Canada; 7 Bridgepoint Collaboratory for Research and Innovation, Lunenfeld - Tanenbaum Research Institute, Sinai Health System, Toronto, Ontario, Canada; Institute of Physiology and Basic Medicine, RUSSIAN FEDERATION

## Abstract

Despite their burden and high prevalence, mental health disorders of children and adolescents remain neglected in many parts of the world. In developing countries, where half of the population is younger than 18 years old, one of every five children and adolescents is estimated to suffer from a mental health disorder. It is then essential to detect these conditions through screening in a timely and accurate manner. But such screening is fraught with considerable ethical, social, and cultural challenges. This study systematically identifies, for the first time, these challenges, along with potential solutions to address them. We report on the results of an international multi- and inter-disciplinary three-round Delphi survey completed by 135 mental health experts from 37 countries. We asked these experts to identify and rank the main ethical, social, and cultural challenges of screening for child and adolescent mental health problems in developing nations, and to propose solutions for each challenge. Thirty-nine significant challenges emerged around eight themes, along with 32 potential solutions organized into seven themes. There was a high degree of consensus among the experts, but a few interesting disagreements arose between members of the panel from high-income countries and those from low- and middle-income nations. The panelists overwhelmingly supported mental health screening for children and adolescents. They recommended ensuring local acceptance and support for screening prior to program initiation, along with careful and comprehensive protection of human rights; integrating screening procedures into primary care; designing and implementing culturally appropriate screening tools, programs, and follow-up; securing long-term funding; expanding capacity building; and task-shifting screening to local non-specialists. These recommendations can serve as a guide for policy and decision-making, resource allocation, and international cooperation. They also offer a novel approach to reduce the burden of these disorders by encouraging their timely and context-sensitive prevention and management.

## Introduction

Children and adolescents (C&A) constitute one-third of the world’s population and half of the population of low- and middle-income countries (LMICs). The vast majority (about 90%) live in the developing world [1]. One-fifth of C&A in LMICs suffers from at least one mental, neurologic, or substance abuse disorder (MNSD), the leading causes of disability worldwide [2–4]. MNSDs represent 13% of the global burden of disease, surpassing both cardiovascular disease and cancer [2,5–7]. They are the major contributor to disease burden in the 15–24 age group and are probably the most neglected aspect of health care [3,8–10]. These disorders account for almost one-third of disability-adjusted life-years (DALYs) lost during the first three decades of life [2,5] and most begin during childhood, adolescence, and early adulthood [4,8,11,12]. In fact, half of MNSDs first appear before age 14, and 75% before age 24, persisting into adulthood and negatively affecting future health and quality of life [2].

Mental health resources for C&A in LMICs are scarce, inadequate, unequally distributed, and inefficiently used, with effective and culturally relevant treatment rarely available [13]. Mental health services in LMICs are 50 times less accessible than they are in high-income countries (HICs) and they tend to be fragmented and very costly. They are poorly integrated into primary health care services and are typically provided at the tertiary level in urban understaffed and crowded institutions [6,14–16].

Nine of ten C&A with MNSDs in LMICs lack adequate care [17,18]. Ninety percent of global mental health resources are limited to the industrialized world, even though over 80% of the world’s population lives in LMICs [19]. This situation is likely to worsen in the coming years, especially in LMICs, due to lack of resources and to the geographic distribution of birth rate growth [18]. Half of the world’s population has access to fewer than one psychiatrist for every 200,000 individuals [17,20,21]. There are, in fact, 748 times fewer psychiatrists in LMICs than in HICs [22]. The "treatment gap" between the demand and the availability of adequate mental health care in LMICs is one of the most pressing ethical issues in these countries [17,23].

C&A mental health care infrastructure is limited by factors such as geography, culture, lack of cost-effectiveness research, and poor intersectoral collaboration [8,21,24,25]. Public expenditure on C&A mental health is negligible in LMICs; most available funds are assigned to the provision of direct clinical services, particularly inpatient care in specialist institutions [7,8,14,17]. This is one of the main reasons MNSDs tend to go undetected and untreated in the developing world. To make matters worse, existing health systems are plagued by poor governance, incompetent leadership, corruption, lack of resources, and undertrained and overworked mental health care workers [11,13,24].

Screening for MNSDs in developing world youth is justified by the large and growing vulnerable population of C&A in LMICs, the high prevalence of untreated or badly treated MNSDs in this cohort [26], the unjust stigmatization and cruel treatment of individuals suffering from these disorders [27–29], and the existence and availability of promising treatments for MNSDs [30,31]. Screening procedures are overwhelmingly recognized as some of the most cost-effective preventive population-wide public health strategies for reducing societal burden and costs related to disease and disability [32–35]. In fact, one of the Grand Challenges in Global Mental Health [20] recommends screening C&A for MNSDs, adapting such screening for local contexts, and incorporating it into primary health care as a part of a core package of services.

Screening enables early identification of at-risk C&A who require urgent attention, intervention, or in-depth assessment. It also leads to timely treatment and reduces the emotional and financial costs of delaying therapeutic procedures or of treating late stages of MNSDs [8,20]. Local screening in LMICs reduces the need for C&A and their families to attend specialized institutions in urban centres just to detect at-risk individuals. This is particularly relevant in contexts in which fragile health systems are overwhelmed with patients in advanced stages of disease [11,12,24,30]. Moreover, screening carried out by non-specialists decreases the total workload of highly trained professionals by enabling them to focus on diagnostic and treatment issues [16].

Screening, however, is laden with ethical, social, and cultural quandaries. When carried out in C&A, it must contend with ethical issues particular to this age group [36,37]. More importantly, most screening instruments and rating scales have been developed in the industrialized world, heavily influenced by the dominant Western biomedical model of mental health that assumes a universal set of causes, diagnostic categories, and treatments [19,38–41]. But this assumption stands on precarious ground, as nosological categories in mental health are based on loose amalgamations of signs and symptoms agreed upon by a consensus of mostly Western mental health professionals [38,42]. The prevailing Western model of MNSDs often collides with the local ways in which these disorders are perceived, understood, and traditionally addressed in LMICs [38,40,41,43,44]. Furthermore, screening, when done without an adequate ethical, social, and cultural framework, may lead to increased stigmatization, discrimination, marginalization, coercive treatment, and exclusion [13,37,45,46].

Screening for MNSDs in C&A living in the developing world is necessary because of the high prevalence and current lack of early detection of these disorders, which leads to a high rate of untreated cases. Given the scant attention to the many context-sensitive issues related to screening; the Western hegemony in the design and validation of screening instruments; the shortage of trained mental health care workers to conduct screening in LMICs; and the unavailability of adequate treatment for at-risk C&A in developing countries, we decided to conduct a three-round international multi- and inter-disciplinary Delphi survey to identify and rank the most pressing ethical, social, and cultural challenges in screening for MNSDs in developing world C&A, and the most suitable solutions to these challenges.

## Methods

We carried out a three-round Delphi survey from October 2013 to December 2014. The Delphi is a structured and systematized consensus-building procedure that uses iterative feedback to collect and distill knowledge from an interdisciplinary panel of experts who remain anonymous to each other [47–50]. This flexible and versatile method has been employed extensively to predict the impact and direction of long-range trends in several fields and to assist in decision-making when designing policies and allocating resources [51,52]. Its main features are anonymity, iteration, controlled feedback, and statistical aggregation of group responses [48]. Heterogeneous groups of experts independently provide responses to focused questions but share decisions through facilitators that collect, organize, summarize, and analyze the responses through formal, systematized mechanisms [49,53]. The systematic nature of the Delphi strengthens the quality, efficiency, and long-term accuracy and validity of panelists’ predictions and judgements [52,54]. The Delphi also avoids challenges and biases inherent to face-to-face meetings and allows group processes when members cannot be physically in the same place.

Based on our experience [20,55–59] and on the existing literature [49,52], we decided that three Delphi rounds using email messages for recruitment, direction, reminders, and for responding to queries, would be sufficient to elicit response stability from our panelists. To ensure a systematic and meaningful synthesis of responses, we drafted, piloted, redrafted, and refined the questions asked of the panelists in every round. We communicated in English with most panelists, and, where needed, in their native languages (mainly Spanish, French, and Portuguese). Periodic email reminders were sent to the experts throughout each round to increase the response rate. We initially pilot tested the questionnaires sent to the experts in each round with a small group of trusted colleagues who fulfilled the inclusion and non-inclusion criteria described earlier, and who would not be incorporated into our panel.

### Panel selection and recruitment

To select our panelists, we sent an email invitation to 490 adult experts from 65 countries who had demonstrated accomplishments and leadership based upon academic, scientific, and professional background and achievements, as evidenced by indexed, high-impact publications, decision-making responsibilities at a local and global levels, or extensive field experience in developing countries. Several of these experts had participated in previous Delphi studies used to identify the Grand Challenges in Non-Communicable Diseases [55] and the Grand Challenges in Global Mental Health [20]. The inclusion criteria for panelist selection are shown in Table 1. We ensured the representativeness of the panel and addressed selection biases by standardizing the expert selection process, using purposive and criterion sampling, and balancing the panel with respect to sex, geographic distribution, and specialty areas. We deliberately included individuals with widely different backgrounds and perspectives in diverse scientific, research, clinical, policy, and advocacy fields with a stake in global C&A mental health. We considered including C&A suffering from MNSDs and their caregivers but decided against it due to the considerable ethical challenges of identifying such a group and the difficulty in obtaining a representative sample.

**Table 1:** Inclusion criteria for the selection of the panelists.

AREA	TYPE OF EXPERT
**Mental, neurologic, and drug addiction and abuse disorders**	Clinicians and researchers Child and adolescent psychiatrists, psychologists, nurses, social workers, and community health workers Social, cultural, and legal psychiatrists, psychologists, nurses, and community health workers Experts in mental health of immigrants, refugees, ethnic communities, and other populations at high risk of stigmatization, marginalization, discrimination, and exclusion Specialists in the design, implementation, monitoring, and evaluation of mental health screening programs and instruments Editors of mental health journals Experts in mental health education, training, and capacity building Teachers with a background in education for special needs students Administrators Executives and managers of local, national, regional, and international mental health institutions Representatives of international mental health societies and associations
**Global and public health**	Clinicians and researchers Public health Epidemiologists Health economists Anthropologists Sociologists Administrators and policy makers Executives and managers of local, national, regional, and global public health institutions and organizations
**Bioethics**	Bioethicists with experience in mental health care Bioethicists focused on the developing world Neuroethicists
**Human rights and the law**	Experts in the rights of children and adolescents Experts in the rights of individuals with disabilities Experts in mental health laws and regulations
**Public and community engagement**	Members of non-governmental organizations (NGOs) and philanthropic organizations related to mental health Community advocates Religious leaders

Panelists were assigned to one of three categories according to their country of origin and their place of work, using the World Bank classification of countries (https://datahelpdesk.worldbank.org/knowledgebase/articles/906519-world-bank-country-and-lending-groups), which is based on gross national income per capita: low-and-middle income country, high-income country, or mixed (if the academic and professional activities of a panelist were spread over a wide range of countries).

### Delphi rounds and data collection and analysis

For Round I, we asked each of the 490 invited experts to identify the three to five foremost ethical, social, or cultural challenges to screening for MNSDs in LMIC C&A, along with three to five potential solutions to address these challenges. In the invitation email, we defined C&A as individuals 19 years old or younger, and screening as a population-wide public health strategy that detects diseases in asymptomatic individuals as early as possible, enabling timely interventions.

Upon receipt, we combined, collated, and analyzed the answers from the experts and organized the challenges and the solutions into two taxonomies subdivided by theme. The systematic line-by-line reading of each response led to the identification of key concepts that became the seeds for the final categories and themes sent back to the panelists for Round II. We used the concepts and themes obtained from previous answers to anchor those that emerged from subsequent responses. As the analysis advanced, an emphasis on and reiteration of certain issues above others became evident; these elements coalesced into frameworks that later gave rise to the final categories and themes. After several manual iterations of this analysis, the final categories and themes emerged. We attempted to make the challenges and solutions independent and mutually exclusive, and relatively exhaustive.

After Round I, we generated a taxonomy of 89 challenges, organized into nine themes, and 93 solutions, arranged into seven themes, for a total of 182 categories. For Round II, this taxonomy was presented to the 165 experts from 40 countries who had completed Round I. Round II was carried out using FluidSurveys.com, now owned by SurveyMonkey.com. We now asked each panelist to select what he or she considered the five most relevant categories for each theme. We then calculated the frequency with which each challenge and solution had been chosen. For Round III, we used the four top categories in each theme, along with those challenges and solutions selected by 50% or more of the respondents. Decisions about numbers and cut-offs were made by discussion and consensus among the authors.

In the email-based Round III, we asked the 135 remaining experts from 37 countries who had completed Round II to do the following three activities: review the final lists of categories, which were grouped into themes and ordered according to the frequency with which they had been selected; re-rank or reject items, if they so wished; and justify their decisions. We compiled the panelists’ comments, linking them to their sex and type of country.

To calculate the final rankings, we assigned scores to categories the following way: for each panelist’s response, the highest ranked category in each section received ***n*** points, where ***n*** = number of categories in the section; the second-ranked category received ***n-1*** points, and so forth. Rejection of a category was translated into a score of “0” (zero). The sum total resulted in the final rankings. We also determined the rejection rate for each category.

### Visual representation of the results

**Fig 1:**
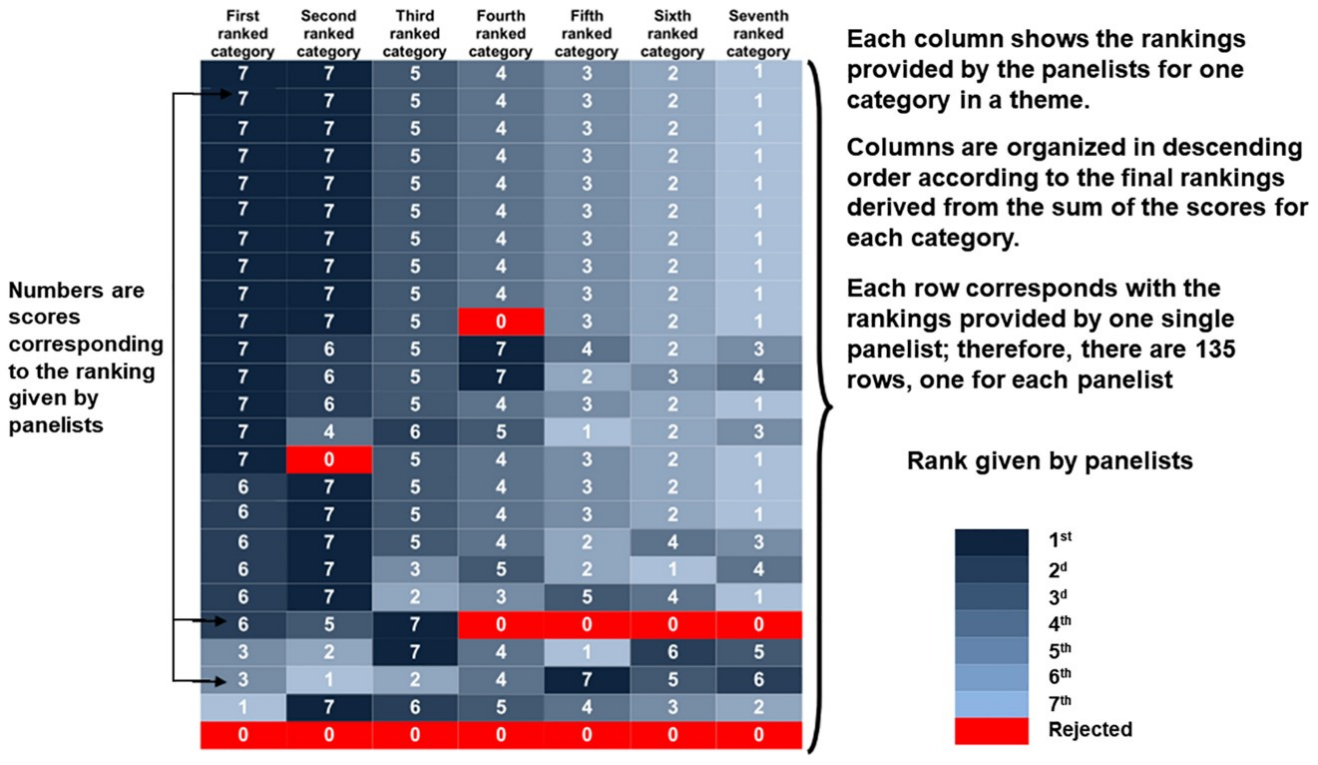
Explanation of the structure of the heat maps and of the correlation between the colour scale and the rankings provided by the panelists. This figure illustrates the general configuration of a heat map, using as an example a theme with seven categories, organized from left to right starting with the category ranked first (the first column), then second, then third, and so on. Values are arranged in decreasing order within each column. Columns and rows are arranged to place similar values together; clusters (blocks of a single colour) show consensus among panelists. Larger values are represented by darker tones and smaller values by lighter tones. Red indicates rejection of a category.

Using the scores for each category, we elaborated heat maps, two-dimensional, colour-shaded graphical representations of a data matrix (the scores from Round III) displayed as a rectangular tiling in which each tile or cell is shaded on a colour scale to represent the value of the corresponding element of the data matrix. Hierarchical clustering on the rows and columns of the matrix results in cells with similar values being placed together, thus generating coherent patterns of colour that provide a logical and synoptic view of our results. This allows for immediate visual assessments and comparisons of the distribution of values. Heat maps provide a logical, coherent, and synoptic view of the results and enable immediate visual assessments and comparisons of the distribution of values without confusing the viewer [60]. We illustrate the structure and logic behind our design of the heat maps in Fig 1.

In the heat maps, the value of a score correlates with the intensity of the colouring of a particular cell. Each row corresponds to the rankings provided by one single panelist; consequently, there are 135 rows. Each column contains the rankings provided by all of the panelists for one specific category. The rows are arranged, first according to type of country, then by sex, and finally, according to the category ranked first (the first column), then second, then third, and so on, with the values arranged in decreasing order within each column. The columns are organized in descending order from left to right according to the final rankings resulting from the sum of the scores for each category. Columns and rows are thus arranged to place similar values together, with clusters showing consensus among panelists.

Colour shows the rank that each panelist gave to each of the categories in a theme. Larger values are represented by darker tones of blue. Red indicates rejection of a category. The sex of the panelists and the type of country to which they were assigned are shown in columns to the right of the rankings. The type of country is indicated by tones of grey, responses from female panelists are shown in light salmon, and those from male panelists are shown in pale green. We chose three colour schemes developed by Brewer *et al*. (http://colorbrewer2.org) that, according to principles delineated by Tufte [61,62], best conveyed differences and correlations among the data.

The protocol of this study was approved by the Health Sciences Research Ethics Board of the University of Toronto Office of Research Ethics (Protocol Reference # 28918). The only incentive to the panelists was the promise of copies of all relevant publications and the assurance that their names would be included in the list of participants who completed all three Delphi rounds.

## Results

Of the 490 experts from 65 countries initially invited to participate, 165 (34%) from 42 countries provided responses to Round I. More than four-fifths of the experts who finished Round I also completed Round II. All 135 experts from 37 countries who completed Round II also finished Round III (Table 2). The members of the final panel appear in the Supporting Information in S1 Table, while the number of experts who completed the Delphi by country is shown in S2 Table.

**Table 2:** Distribution of the experts in each Delphi round, by sex and type of country according to World Bank classification of nations.

		Invited	Responded to Round I		Responded to Round II		Responded to Round III	
		*n*	*n*	*%*	*n*	*%*	*n*	*%*
**Females**	**HIC**	86	27	31	20	74	20	100
	**LMIC**	104	33	32	29	88	29	100
	**L / H**	6	3	50	2	67	2	100
**Males**	**HIC**	135	39	29	29	74	29	100
	**LMIC**	141	55	39	47	85	47	100
	**L / H**	18	8	44	8	100	8	100
**TOTAL**		**490**	**165**	34	**135**	82	**135**	100

**HIC**, High-income countries; **LMIC**, Low- and middle-income countries; **LMIC / HIC**, mixed.

**n** = number of experts in each category.

**%** = percentage of experts in each category with respect to those in the same category in the previous Round.

The composition of the panel did not vary significantly across the Delphi rounds in terms of sex, geography, type of country, or expertise. We compared the distribution by sex, country, and field of work of the 165 experts who completed Round I to that of the 325 individuals who were invited and chose not to participate in our study. The distribution by sex and country for this Round is noted in Table 2, and the comparison between the numbers and percentages of respondents and non-respondents to Round I appears in S3 Table (found in the Supporting Information). With the exception of the first two categories, it is clear that the proportion of responders / non-responders is quite similar. The higher percentage of responders in these two top categories is not unexpected, as experts who specialize in mental health of vulnerable populations, global mental health, public health, epidemiology, or health economics, would be more likely to show interest in the topic of our study, and to feel sufficiently knowledgeable to respond to the questions.

### Challenges and solutions

After Round I, we organized the proposed 89 challenges into nine themes, and the 93 potential solutions into seven themes. By the final Round, the panelists had selected and ranked 39 challenges, grouped into eight themes, and 32 potential solutions to these challenges, arranged into seven themes. These challenges and solutions are shown in Table 3. Given that the theme “Public and community engagement” only had five challenges, and that, in Round II, panelists were asked to choose exactly five categories for each theme, we decided to combine these five challenges with those of the theme “Education, training, and capacity building”, thereby reducing the number of separate challenge-related themes from nine to eight.

**Table 3:** Comparison and correspondence between the final challenges and their potential solutions.

The challenges	The solutions
**C-I. Inadequate design and validation of the screening tools**	**S-I. Adequate design and validation of the screening tools**
Screening tools and approaches based on Western understanding and assumptions related to MNSDs may miss local indicators of disease, or may mislabel and pathologize normal or culturally accepted behavioural variations Psycho-socio-cultural differences within and among countries may not be taken into account in the design of the screening tools Screening tools and rating scales may lack evidence-based design, validation, normalization, and standardization Screening tools may lack adequate sensitivity and specificity given the complex and multi-dimensional nature, etiology, and clinical presentation of MNSDs	Develop affordable and scalable screening tools that are easy to understand and simple to administer and that have clear definitions, precise goals, and a well-structured response set Ensure that the screening instruments and rating scales are flexible, evidence-based, culturally and socially valid (focusing on languages and educational levels), and age-appropriate, but standardized to enable cross-cultural comparisons Pilot the screening instruments in relevant local communities Ensure that the screening instruments not only evaluate symptoms but also provide health workers with sufficient information to help at-risk individuals
**C-II. Inadequate application and interpretation of the screening tools**	**S-II. Adequate application and interpretation of the screening tools**
Screening in schools will miss individuals who do not attend educational institutions Screening may be hampered by inaccessibility, cost of transportation, unawareness about the location of the screening, or inconvenient timing Families may wrongly believe that the more screening questions they answer affirmatively, the higher the likelihood of receiving medical, educational, or financial support The screening tools may not be applied or interpreted correctly, particularly if administered by non-specialists or lay people who may make inadequate judgments Adults may fear that intrusive questions about sex or drug use may lead to these behaviours in the individuals screened	Use qualitative research to determine local views and beliefs on MNSDs such as age-appropriate behaviour and idioms of distress When the instrument needs translation, recruit experienced translators, validate translations through bilingual experts and native speakers of the local language, and use extensive back-translations Choose an accessible, socially and culturally acceptable setting for the screening Use vignette-based descriptions and pictorial explanations to describe difficult concepts Consider using, where appropriate, socially- and culturally-sensitive computer, web-based, and mobile screening tools
**C-III. Weaknesses of the screening program**	**S-III. General logistical issues related to the screening programs**
A badly designed, locally-inappropriate screening program may do more harm than good Marginalized populations may not be screened Screening programs may underestimate the contributory causal role of psychosocial determinants of MNSDs such as poverty and all of its various consequences Screening programs may be geared towards the needs of the screeners or researchers, and not of those screened Insufficient evidence exists in LMICs that screening for MNSDs is reliable, cost-effective, or harmless Screening programs may overlook important environmental contributors to MNSDs such as iodide deficiency, tuberculosis, malaria, HIV / AIDS	Coordinate the screening programs with general health, education, and social services, and with NGOs and philanthropic institutions Provide adequate explanations to those screened and their families about MNSDs, the screening process, and available therapeutic resources and follow-up, to maximize benefits to at-risk individuals Establish long-term, recurrent screening programs rather than one-off exercises To build trust, incorporate relevant stakeholders at all stages of the screening programs
**C-IV. Lack of a pre-existing mental health system within the national public health infrastructure**	**S-IV. General logistical issues related to the mental health care infrastructure**
Screening for MNSDs will not be beneficial if it is not integrated into the primary health care system Screening programs may be hampered by the lack of a national mental health strategic plan that provides adequate funding, administrative and logistic support, and supervision Screening for MNSDs may lack political support and may not be perceived as urgent compared to other priorities by uninformed decision-makers Mental health services in some geographical regions may not be geared towards children and adolescents	Incorporate screening programs for MNSDs into community-based primary health care and consider integrating them with public health initiatives Ensure that post-screening accessible, affordable, and culturally and socially appropriate treatment and follow-up are available, including early interventions and psychological first aid Facilitate family-, school- and community-based interventions that emphasize resilience and cognitive and emotional skills Only carry out screening for MNSDs if adequate diagnosis, treatment, and follow-up are available
**C-V. Stigmatization, discrimination, marginalization, and exclusion**	**S-V. Human dignity, human rights, privacy, consent, confidentiality, and legal and regulatory considerations**
At-risk individuals, their families, or their communities can be stigmatized, discriminated against, marginalized, or subjected to exclusion and violence Respondents may withhold information out of fear of stigmatization and discrimination for appearing indiscreet or vulnerable (particularly in the case of males) The screening program may be hindered by social, cultural, or religious biases Parents and caregivers of at-risk individuals may be subjected to “blaming and shaming” Individuals who screen positive may suffer exclusion from health services, educational institutions, or jobs	Ensure that screening programs are carried out within a human rights framework that respects the dignity of those screened Anticipate, prevent, and reduce stigmatization, discrimination, marginalization, and neglect of screened and at-risk individuals Guarantee the privacy and confidentiality of all information related to screened individuals and their families, making all concerned aware of this protection Make sure that every screened child and adolescent is regarded as having equality before the law, and that their rights are respected post-screening throughout the mental health system Obtain informed consent or assent for the screening and follow-up of the individual screened without coercion
**C-VI. Fear and lack of trust**	
Social desirability bias may prevent candid or truthful responses Illiteracy or lack of education may increase suspicion and unwillingness to participate in the screening Screening may be perceived as too intrusive Individuals may not trust screeners, health care workers, or Western medicine in general If screening is performed or organized by foreigners it may create resentment Parents and caregivers who have themselves suffered from MNSDs and who have been treated cruelly may refuse to have their children screened Parents and caregivers may encourage those screened to under-report MNSD symptoms for fear that their children will be taken away and institutionalized	
**C-VII. Human dignity, human rights, privacy, autonomy, consent, and confidentiality**	
Screening programs may not be able to handle the complexities of consent (such as age of autonomy), information sharing with parents and others, and data management (access, storage, protection, and destruction) Free and informed consent of children and adolescents may be ignored Individuals may feel vulnerable in the absence of a guarantee of privacy and confidentiality Genuine informed consent may be compromised by lack of knowledge about MNSDs or by inadequate explanations about the screening process	
**C-VIII. Education, training, capacity building, and public and community engagement**	**S-VI. Education, training, and capacity building**
In most LMICs, there is a severe shortage of specialized mental health care workers and of adequate specialization programs Already overburdened health care workers and teachers may be unmotivated to participate in the screening programs and may resent the encroachment on their everyday duties Caregivers without adequate guidance and support may be unprepared to address the needs of children and adolescents identified as at-risk through the screening In low-resource and low-education settings, properly sensitizing, training, and supervising local non-specialists may be very challenging	Provide adequate training, support, and supervision for screeners, making them aware of their own beliefs and biases Train community health workers, especially in remote areas, to carry out screening and affordable and cost-effective primary mental health interventions Include mental health as an integral part of academic curricula at all levels of education Provide adequate training, support, and supervision of all health workers concerned with the care and follow-up of at-risk individuals and their families
	**S-VII. Public and community engagement**
	Increase the profile of MNSDs by incorporating them into general public health education campaigns Design and carry out at regular intervals culturally-sensitive, adaptable, and empowering public engagement strategies that raise awareness, provide information, address fears, and build trust Engage local communities in respectful dialogue that critically examines the potential benefits and drawbacks of screening programs Identify and engage influential, high-profile local individuals, especially if they have successfully overcome MNSDs, who are willing to participate in public engagement campaigns Consider the use of engagement strategies such as community meetings, home visits, posters, song, dance, and theatre, building upon lessons and experiences from similar previous initiatives Before implementing screening programs for MNSDs, determine the baseline acceptance, motivation, and support for them in target communities

**Fig 2:**
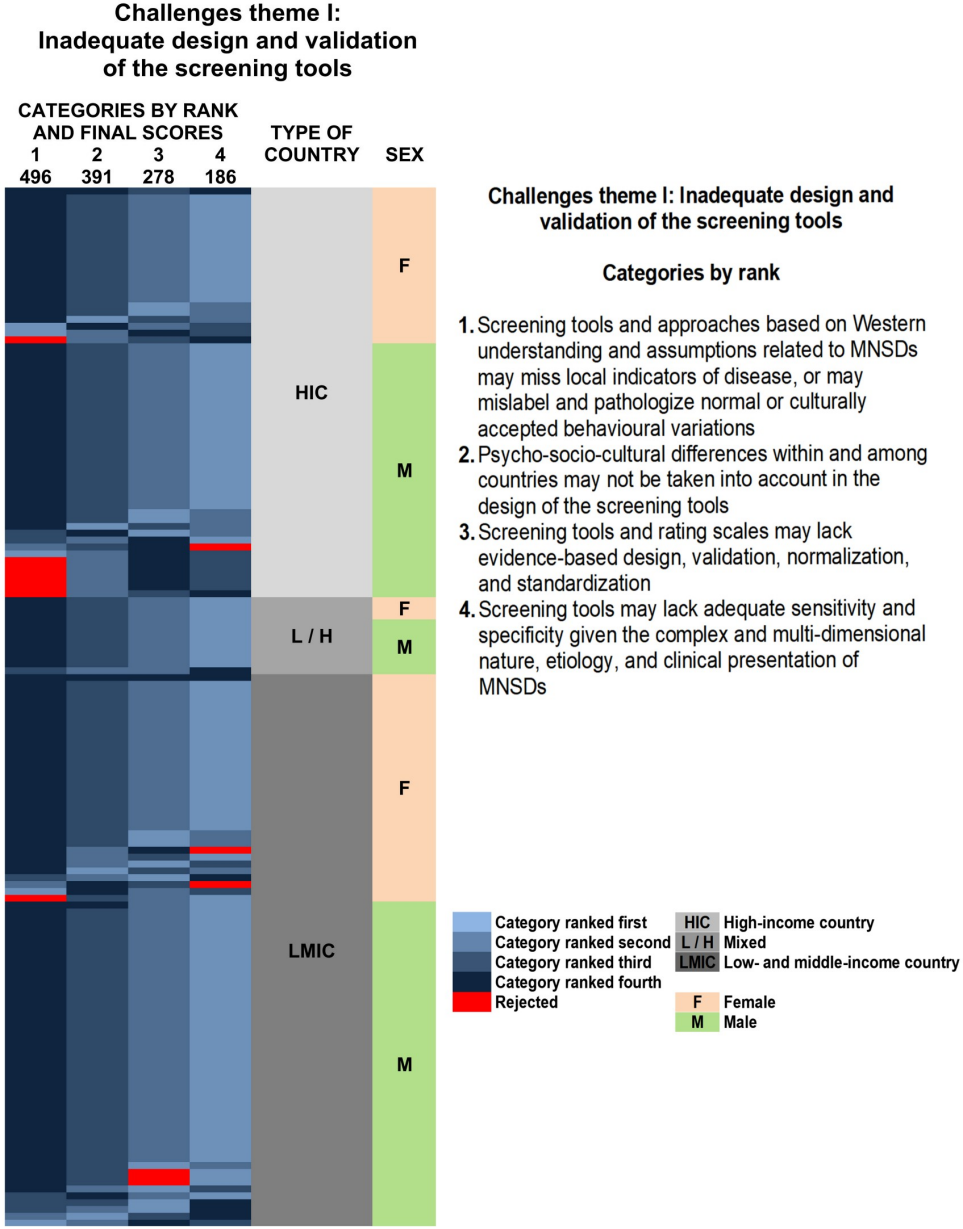
Heat map with the distribution of the final rankings of challenges theme I: Inadequate design and validation of the screening tools identified by the panelists by sex and type of country according to the World Bank classification. The rows are arranged, first according to type of country (HIC, L / H, LMIC), then by sex (F, M), and, finally, according to the category ranked first (the first column), then second, then third, and so on, with the values arranged in decreasing order within each column. The columns are organized in descending order from left to right according to the final rankings resulting from the sum of the scores for each category; the numbers at the top of each column correspond to these totals. Columns are therefore arranged to place similar values together; clusters (blocks of a single colour) show consensus among panelists. Larger values are represented by darker tones. Red indicates rejection of a category. The sex of the panelists and the type of country to which they were assigned are shown in columns to the right of the rankings. The type of country is indicated by tones of grey, responses from female panelists are shown in light salmon, and those from male panelists in pale green. In contrast to Fig 1, which explains the design of the heat maps, in this illustration the numerical values of the scores are not included because the tone of the colour clearly conveys this information.

As mentioned in the Methods section, we created 15 heat maps to facilitate the visualization of the results of our Delphi. We show here the heat map corresponding to the top ranked challenges of the first theme, “Inadequate design and validation of the screening tools” (Fig 2). The other 14 heat maps can be found in S1 Fig of the Supporting Information, with S1a–S1g Fig showing the heatmaps for the challenges, and S1h–S1n Fig showing those for the solutions. In all the heat maps, large blocks of the same colour indicate consensus regarding the ranking of a category. The numbers representing the scores given by the panelists to each challenge or solution do not appear in each cell, as the colours and their intensity convey the same information.

**Fig 3:**
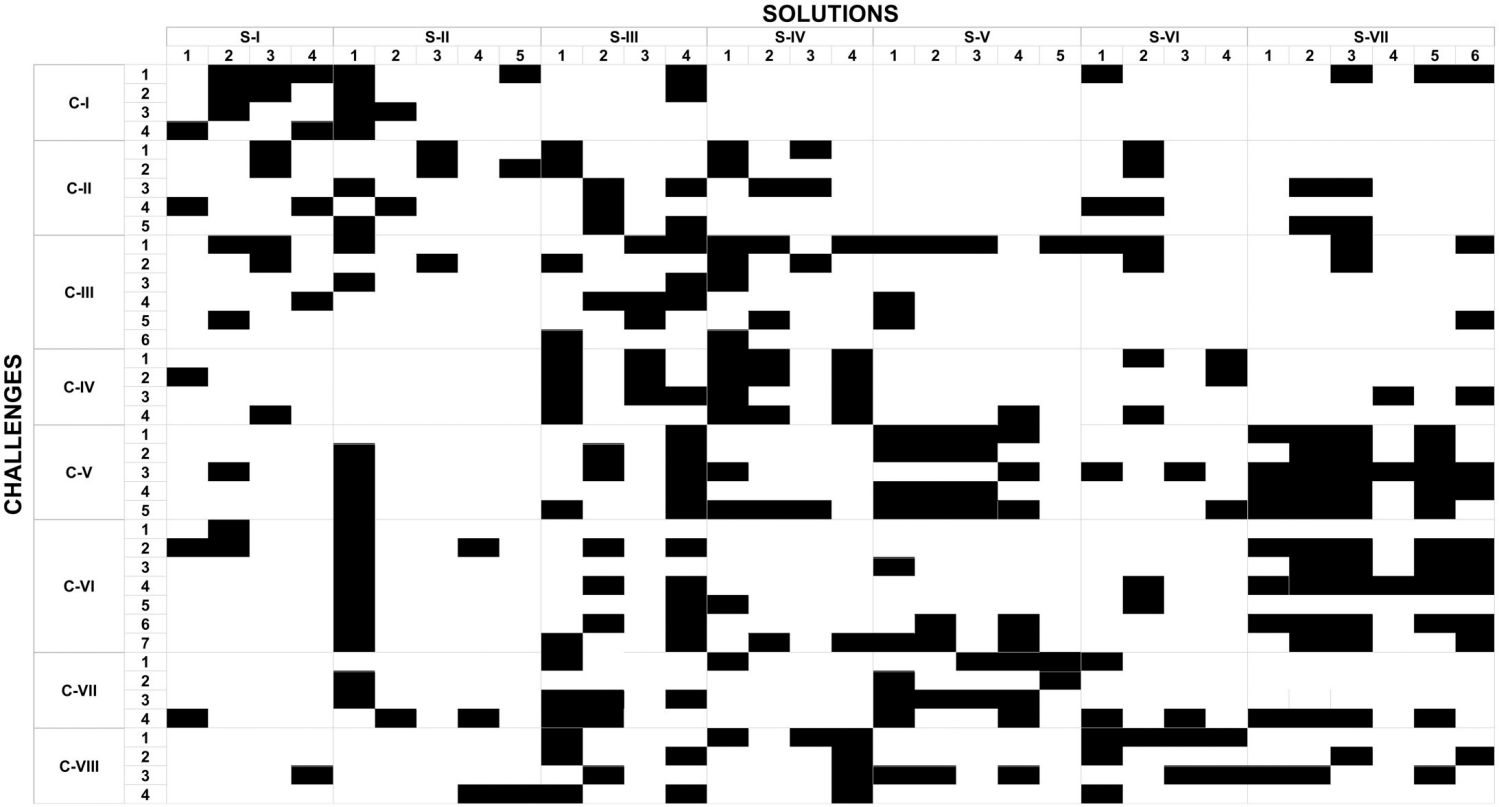
Correlation between the final challenges and solutions. The challenges are organized by theme along the vertical axis and are indicated by C-I to C-VIII, with Arabic numerals corresponding to the challenges within each theme, by rank. Similarly, the solutions appear organized by theme along the horizontal axis and are indicated by S-I to S-VII, with Arabic numerals corresponding to solutions within each theme, by rank. In this rectangular binary matrix, a black cell indicates that a particular solution can meet a specific challenge. Large vertical blocks of black cells show that a solution is relevant to several challenges, while large horizontal blocks represent challenges that can be addressed by several potential solutions.

Fig 3 shows the correspondence between challenges and solutions. All challenges could potentially be addressed by at least 2 solutions, and all solutions could potentially address at least 3 challenges. Challenges were addressed by 2 to 17 solutions, and solutions could potentially address 3 to 21 challenges.

Engagement with the topic of our study was evidenced by comments provided by the panelists regarding the themes, the categories, and their rankings. The themes that received the highest number of comments were mental health care infrastructure; inadequate design, validation, application and interpretation of screening tools; fear and lack of trust; and human dignity, human rights, privacy, autonomy, consent, and confidentiality. Interestingly, LMIC panelists provided more comments than those from HICs, which could indicate a greater investment with the subject matter.

The low rates of rejection of the categories strengthen the final consensus. Even the most rejected category was eliminated by less than 10% of the panelists. In the heat maps, the rejection of all the categories in a given theme appears as a single horizontal red line. Only 5 panelists, all from the developing world, made across-the-board rejections, another indication of strong feelings about the topic of our study.

## Discussion

Ours is the first systematic identification and prioritization of ethical, social, and cultural issues pertaining to screening for MNSDs in developing world C&A. Our results echo several of the proposals of the Grand Challenges in Global Mental Health [20], in particular the need to adapt screening to local contexts, and its incorporation into primary health care.

### Design and validation of screening tools

The panel recognized the challenges involved in the epidemiological justification, logistics, long-term perspective, and cost-effectiveness of initiating screening programs with instruments appropriate to local needs. The experts agreed that all tools used must be valid, reliable, accurate, and locally acceptable in order to enhance their benefits and reduce their potential harms; this view agrees with the literature [63,64]. Panelists emphasized the need for adequate post-screening referral and follow-up of C&A found to be at-risk [34,35,65]. Our panel noted that the validity and reliability of screening tools depend on the soundness of the nosological taxonomies on which they are based [66]. However, some experts believed that screening should be less about capturing an ideal disease construct and more about uncovering distress that can be addressed by effective care.

Too often, screening tool development is biased by external agendas irrelevant to the task at hand [23,37,67]. Also, the requirements for permissions and payment of instrument licensing fees are unrecognized challenges for LMICs.

The experts warned that instruments that yield false negatives could lead to lack of adequate treatment, while those that yield false positives could lead to unjustified medicalization and cruel stigmatization of those erroneously found to be at risk [66,68–70]. Other challenges mentioned by our panel were language difficulties, the misleading capture of transitory rather than long-lasting states of mind, the expected reluctance to report symptoms deemed to be embarrassing, and the possibility of inaccurate interpretation of screening results, considerations that have been alluded to in previous literature [71–73], but have been emphasized more strongly by our panelists.

Our results challenge the premise that it is possible to design a universally applicable screening tool that can be used for C&A across the world. Thomas Achenbach and his group have developed screening instruments that have been used in 44 societies on five continents and have been translated into 85 languages [74–78]. These tools are based on a classification of societies using an omnicultural mean from which a local set of norms can be developed. In Achenbach’s group experience, cultural differences do not result in significant discrepancies when using these instruments. This research group believes that empirically-based assessments with standardized tools and procedures offer a cost-effective way to identify problems in C&A of different cultural backgrounds [74]. Our results, in agreement with those of others [72,79,80], contradict the idea that “universal” screening instruments can be used in contexts very different from those in which they were designed. Our panelists stress the point that current screening tools that have been developed and validated in HICs under Western paradigms are liable to miss local indicators of disease, may mislabel and pathologize normal or culturally acceptable behavioural variations, or may ignore psycho-socio-cultural differences within and among geographic regions.

### Adaptation and translation of screening tools

In line with the concern regarding the use of “universal” screening tools, the panel shared with others [38,40,41,81] an acute awareness of the Westernization of currently used screening instruments. Our experts recommended that all instruments used in LMICs be context-sensitive, evidence-based, age-appropriate, easy to administer, reliable, affordable, and cost-effective [20,65,68,82]. They pointed out that several such instruments are currently used in the developing world [63,83–87]. At the same time, it was not lost on the panelists that adequate resources and technical expertise to design locally appropriate questionnaires may not always be available. Thus, in some instances, appropriately adapting and translating well-validated standardized questionnaires could be the practical and cost-effective solution [16,88–90]. This is especially so because standard methods and guidelines have been developed to increase concordance between the adapted and translated instruments and their originals [66,82,91,92].

### Application of screening tools

The panel considered the accessibility and acceptability of locations in which screening might best be conducted, along with the timing of the application of the instruments. While screening in schools is favoured by many [93–95], the experts drew attention towards the needs of marginalized C&A, who may not attend educational institutions. Screening that includes interviewing multiple key informants [96–98] was recommended. Panelists praised the use of vignettes, pictorial representation, and socially- and culturally-sensitive electronic screening tools and post-screening interventions previously shown to be effective in LMICs [99–105].

### Integration of screening into primary health care

Our experts agreed that screening for mental health, along with follow-up of at-risk C&A in LMICs, needs to be incorporated into community-based primary health care. This view coincides with a major recommendation of the Grand Challenges in Global Mental Health [20,106]. Integrating C&A mental health programs such as screening into primary health care has been shown to be effective, accessible, affordable, and family-friendly [24,71,107–113]. Such integration ensures that C&A with MNSDs are treated as all other medical patients, thus minimizing stigmatization [17]. Our panelists considered that successful screening of C&A in primary care could lead to a surge in demand for mental health treatment in contexts where specialists are scarce or non-existent. However, this issue may subside once primary health care workers gain experience in the care of at-risk individuals [107,109,110].

### Need for post-screening follow-up

In line with canonical screening practices [35,114,115], most panelists agreed that screening should only be carried out where post-screening follow-up is available. They considered it would be unethical to burden C&A and their families with positive screening results in the absence of access to effective treatment. In our experts’ view, a culturally-sensitive national mental health strategic plan that provides adequate long-term funding, administrative and logistic support, and supervision of screening endeavours and treatment facilities must be in place before instituting screening. Our experts also advocated for a mental health subsystem focused specifically on C&A with a functioning system of referrals and contra referrals, adequate insurance coverage, support for families, communities, and teachers, and political backing. These prerequisites have been brought up in the literature [11,17,24,116,117]. Demanding such provisions before the start of screening was the issue that generated the greatest discrepancy among the panelists. In their comments, dissenting experts, mostly coming from LMICs, stated that such requirements could easily become an excuse for inaction. In their view, the burden of C&A MNSDs and the existence of effective interventions (even when not locally available) justified screening. They argued that the identification of large numbers of individuals needing mental health care constitutes a powerful source of pressure on governments, charitable organizations, pharmaceutical companies, private donors, and global institutions to build new needed resources.

### Scaling-up of existing screening programs

Our panel noted that, while successful and cost-effective screening initiatives do exist in LMICs, most need to be scaled-up. The literature indicates that achieving this goal requires a long-term vision, flexibility, and political support [7,11,118,119]. The sustainability of scaling-up initiatives depends on sufficient and stable funding from many quarters: national health budgets, the local private sector, and global foundations and financial institutions [79,120]. Panelists were confident that, in the long term, the costs of providing care to at-risk C&A would be offset by a reduction in the burden of disease due to screening and early treatment.

### International collaborations

The panel strongly endorsed long-term, sustainable South-South and North-South collaborations aligned with local health-care needs. Such partnerships help prioritize C&A mental health issues in LMICs, provide mentorship, mobilize political will, and shape policy development. Existing successful collaborations [121–125] and international consortia [79] are available to provide leadership. Our findings can result in the formation of more such collaborations, for instance the creation of an online repository of locally developed and validated screening tools and of ongoing results of LMIC screening programs.

### Ethical and human rights issues

The panel recommended a human rights framework for screening. Our experts defined ethical screening as embodying respect for the dignity of all screened C&A. They noted that requirements for free, uncoerced, and informed consent and assent needed to be based on participants’ maturity, age of autonomy, and legal competence. The conflict between Western interpretations of individuality and autonomy, and the reliance on collective decision-making common in some LMICs elicited their attention. Communal approaches to privacy, autonomy, and confidentiality, and marked gender inequalities, may lead to decision-making that marginalizes the opinions of C&A (particularly girls) regarding their mental health care.

Fundamentally, however, screening needs to ensure privacy and confidentiality, allow information sharing with caregivers, and guarantee regulation of access, storage, protection, and destruction of screening data to prevent information misuse [13,17,23,36,37,45,126–130]. The panelists noted the importance of attitude and demeanor on the part of Western advisors of screening programs in LMICs, particularly the avoidance of what could be interpreted as condescension, disapproval, or arrogance. They also pointed out, as has been done by others, that screening must focus on the needs of the C&A and not on the personal, academic, or professional interests of the screeners [37,79].

### Fear and lack of trust

Myths and misconceptions about MNSDs can arouse suspicion and resentment towards screeners, screening programs, health administrators, and Westerners, thus discouraging participation [17,23,37]. As most developing countries endured the experience of colonization, the rejection against Western cultural concepts and constructs of mental health is understandable [38,41,44]. Screening tools have been developed for and validated overwhelmingly in adult populations in industrialized countries. Caucasian populations, cultures, and societies have been considered normative, with the “culture-specific” label reserved for those of non-Western origin. Some panelists wondered whether the legacy of colonialism in LMICs could have forever sabotaged the participation of foreigners from HICs in the screening programs.

The panel noted that C&A may be reluctant to participate in screening, especially if they feel coerced by adults. In turn, adults may fear that the screening will induce socially unacceptable behaviours in their children or that those at-risk will be taken from them. Conversely, they may expect to be provided with educational or financial support if they have a child at risk, becoming disappointed when this proves untrue. Screening may be perceived as overly intrusive. Informants may fear appearing indiscreet or vulnerable, not knowing what information to disclose and what to keep within the family. Assurances of confidentiality need to be guaranteed by trusted elders.

### Education, training, and capacity-building

Our panel consensus, in line with WHO recommendations [22,131–133], advocates for the design and implementation in LMICs of socially- and culturally-sensitive education and capacity-building strategies that include training, support, and supervision for all screening staff [16,20,24,134–136]. These strategies encompass “task shifting” and devolving responsibilities, when appropriate, to non-specialists or lay individuals [137–139]. They also include community-based care, clear referral pathways, and compensation / recognition commensurate with the importance of the work [16,24,134]. The panelists noted that C&A mental health care providers in LMICs are currently overworked, overwhelmed, and underpaid, and most feel unsupported. LMICs can benefit from South-South and North-South collaborative capacity-building efforts [8,11,24], especially those that encourage “brain recirculation”, harnessing the talents of the diaspora of mental health care workers from LMICs now living and working in HICs [16,20,134].

### Public and community engagement

The experts stressed that baseline local acceptance and support for screening in target populations was required before the roll-out of any screening program. Incorporating relevant stakeholders at all stages of the program builds trust, integrates system-level considerations with community-level needs, and helps address social and cultural divergences and misunderstandings [20,140]. Periodic, culturally sensitive, and empowering public and community engagement strategies implemented in parallel with screening initiatives considerably strengthen support for the screening programs. Even though these strategies may unrealistically raise expectations, on the positive side, they raise awareness about C&A MNSDs, provide information, address concerns, increase a sense of ownership of the programs, and decrease stigmatization [141,142]. Existing manuals such as the one developed by the Child Mental Health Awareness Task Force [143], serve as potential guides to such initiatives.

### General considerations on the panelist consensus

Three general patterns common to the 15 heat maps of this study (Fig 2 and S1 Fig in the Supporting Information) emerge from our findings: categories ranked first or second show greater consensus than lower ranked ones; the patterns of consensus (or disagreement) tend to remain constant regardless of the type of country; and male respondents from both HICs and LMICs tend to disagree more among themselves than female respondents, especially with regard to solutions.

### Future avenues of development

Our findings suggest the following specific courses of action in accordance with the global mental health literature [18,120,142,144–147]. Research is needed on local social and cultural issues that affect the screening programs, their integration into primary health care, and their scaling-up. The discovery of biomarkers of neuropsychiatric illness will, in time, make screening more accurate and the nosological taxonomy of MNSDs perhaps less culture-bound. Insurance coverage for mental health screening and subsequent treatment and follow-up is an urgent necessity everywhere. The sharing of experiential knowledge among LMICs will streamline and speed the design and implementation of screening programs. In the next few years, technological advances in artificial intelligence will facilitate analysis of increasingly large amounts of screening data. We propose that sections on C&A mental health screening be included in the guidelines of relevant professional societies. Importantly, our results underscore the need for policies, laws, and regulations that support screening programs and that align with United Nations human rights instruments and standards [17,130,132,148–151]. This requires securing political commitment, actively involving relevant stakeholders (including “champions”, local high-profile individuals), and taking advantage of “policy windows” [133,152–155]. Screening initiatives need to ensure that at-risk C&A receive the same legal protection as individuals suffering from other health conditions. In our view, informed by the findings of this study, the severe burden of C&A MNSDs justifies embedding screening as a bedrock program in WHO action plans [131] and including it among the Sustainable Development Goals. WHO core packages such as the Mental Health Gap Action Programme (mhGAP) [156] can help involve non-specialists in screening programs, integrate screening into primary health care, and guide the scaling-up of screening initiatives [150]. WHO documents [157] are available to guide the development of policies and norms governing screening programs in LMICs. The WHO can also catalyze the inclusion of screening initiatives in international collaborations by providing incentives and support. It is well placed to help overcome political barriers, share successful screening experiences, and guide program and post-screening monitoring [6,79].

### Strengths and potential limitations of the study

The Delphi method and the multi- and inter-disciplinary global panel of experts we recruited gives credence to the validity, reliability, and accuracy of our results, as does the panel’s relative stability over three rounds. Panel diversity was a strength. The sex ratio was almost equal, and throughout the three rounds, more than half of the experts were from LMICs. The drop-out rate mirrored that of other Delphi studies [48,158]. The high response rate in Rounds II and III reinforces the validity of the solutions proposed. As with all Delphi surveys, a different panel might have arrived at somewhat different results. Our findings reflect the opinions of a particular group of individuals at a specific point in time. However, the generalizability of our results is strengthened by the expertise and representativeness of our panelists and the concordance of many of their views with published literature. While our study took place in 2014, circumstances regarding mental health have changed little in the developing world, so we consider that our findings continue to be relevant. Panelist attrition might have influenced the results, but the final panel closely mirrored the original group of invitees. In terms of the configuration of our Delphi panel, it could be argued that some LMIC experts belong to local elites and are unaware, perhaps, of all the needs of disadvantaged C&A with MNSDs in their countries. We have no evidence that this is the case. While we could have included in the Delphi panel C&A suffering from MNSDs as well as their parents or caregivers, we would have faced considerable ethical challenges related to consent and assent. Absent or unreliable internet access could have limited the participation of some experts. Still, we did manage to exchange emails with panelists from some LMICs with relatively poor internet service. Non-native English speakers, especially in LMICs, may have excluded themselves. However, while communications with the panel were largely in English, we also managed to correspond with some LMIC panelists in their native languages. The challenges and solutions identified by the panel could hypothetically differ as applied to specific MNSDs, but this possibility was not mentioned by any of the panelists. Shared risk factors, overlapping symptoms, and co-morbidity have favoured grouping diverse MNSDs together [159] despite acknowledged brain differences among them [160,161] and variation in genetic risks [162].

## Conclusions

Rigorously selected expert panelists were asked to identify challenges and solutions to ethical, social, and cultural issues related to screening for MNSDs in C&A of the developing world. Specific solutions proposed by the panel include the incorporation of screening into primary health care, the implementation of education and capacity-building strategies, and the design of optimal policy frameworks. Our results provide an evidence-based foundation for policy- and decision-making, priority-setting, resource allocation, and international cooperation in C&A mental health. At the same time, we highlight some interesting disagreements between experts from LMICs and HICs on the use of Western versus locally developed screening tools, on the roll out of screening programs in the absence of accessible treatment, and on the question of the relationship of screening to the stigma that attaches to mental health. These important issues await further exploration. We hope that our results can contribute to improve the mental health and quality of life of the vast number of vulnerable children and adolescents in the developing world.

## Supporting information

S1 Table Final list of panelists. (DOCX)

S2 Table Number of panelists who completed all rounds of the Delphi, by country. (DOCX)

S3 Table Number and percentage of respondents and non-respondents to Delphi round I, by field of expertise. (DOCX)

S1 Fig A-N. Heat maps for challenges themes II-VIII and solutions themes I-VII. (DOCX)
