# Supplementary material for: The ethical, social, and cultural dimensions of screening for mental health in children and adolescents of the developing world
Source: PLoS One. 2020 Aug 24;15(8):e0237853. doi: 10.1371/journal.pone.0237853 (PMC7446846; doi:10.1371/journal.pone.0237853)
Supplement: S1 Table — (DOCX) [file pone.0237853.s001.docx]

**Supporting Information**

# Final list of panelists

Lameze Abrahams

*South Africa*

Thomas Achenbach

*United States*

Colleen Adnams

*South Africa*

Alastair K Ager

*United States*

Sergio Aguilar-Gaxiola

*Mexico / United States*

Lilia Albores-Gallo

*Mexico*

Atalay Alem

*Ethiopia*

Laura Helena S G de Andrade

*Brazil*

Narendra K Arora

*India*

Florence Baingana

*Uganda*

Muideen Owolabi Bakare

*Nigeria*

Myron Lowell Belfer

*United States*

Gary S Belkin

*United States*

Arvin Bhana

*South Africa*

Thomas Boat

*United States*

Paul Anthony Bolton

*United States*

Isabel Altenfelder Santos Bordin

*Brazil*

Ulla Botha

*South Africa*

Jonathan Burns

*South Africa*

Ines Bustamante-Chávez

*Peru*

Jorge Caraveo-Anduaga

*Mexico*

Santosh K Chaturvedi

*India*

Ana Cristina Chaves

*Brazil*

Daniel Chisholm

*United Kingdom / Switzerland*

Neerja Chowdhary

*India*

Pamela Y. Collins

*United States*

Francine Cournos

*United States*

Füsun Çetin Çuhadaroğlu

*Turkey*

Petrus De Vries

*South Africa*

Mary Desilva

*United Kingdom*

Djibo Douma Maiga

*Niger*

Stefan Du Plessis

*South Africa*

Madeleine Duncan

*South Africa*

Julian Eaton

*Nigeria*

Rebecca Elliott

*United Kingdom*

Mayada Elsabbagh

*Canada*

Eddy Eustache

*Haiti*

Marcelo Pio de Almeida Fleck

*Brazil*

Gregory Fricchione

*United States*

Judith Friedland

*Canada*

Daniel Shuen Sheng Fung

*Singapore*

Michelle Funk

*Switzerland*

Octavio Gárciga-Ortega

*Cuba*

Camila Gianella- Malka

*Peru / Norway*

Anna Soledade Graeff-Martins

*Brazil*

Rebecca Freeman Grais

*France / United States*

Thomas Grisso

*United States*

Oye Gureje

*Nigeria*

Charlotte Hanlon

*United Kingdom*

Sue Hawkridge

*South Africa*

Andreas Heinz

*Germany*

Gerhard Heinze

*Mexico*

Melanie Hendricks

*South Africa*

Helen Henningham

*Jamaica / United Kingdom*

Madelyn Jane Hsiao-Rei Hicks

*United Kingdom*

Rosa Hoekstra

*Netherlands / United Kingdom*

James C. Hospedales

*Trinidad and Tobago*

Daniel Mwesigwa Iga

*Uganda*

Thomas R. Insel

*United States*

Ahmad Jalili

*Iran*

Bhoomikumar Jegannathan

*Cambodia / Sweden*

Mark Jordans

*Netherlands / United Kingdom*

Sylvia Kaaya

*Tanzania*

Craig Katz

*United States*

Sudhir K Khandelwal

*India*

Lincoln L. Khasakhala

*Kenya*

Christian Kieling

*Brazil*

Ranga K. Krishnan

*India / Singapore*

Roberto Lewis-Fernández

*United States*

Elizabeth Lin

*Canada*

Fernando Lolas-Stepke

*Chile*

Carmen López-Stewart

*Chile*

Fred Lowy

*Canada*

José Lumerman

*Argentina*

Crick Lund

*South Africa*

Athena Madan

*Canada*

Edah Wangechi Maina

*Kenya*

Jair De Jesús Mari

*Brazil*

Ana Cecilia Petta Roselli Marques

*Brazil*

Helen McConachie

*United Kingdom*

Kwame McKenzie

*Canada*

Marcelo Feijó De Mello

*Brazil*

Eurípedes Constantino Miguel

*Brazil*

Fareed Aslam Minhas

*Pakistan*

Isaac Mohan

*India / Australia*

Andrew Mohanraj

*Malaysia*

Amrie Morris-Patterson

*Saint Vincent and The Grenadines*

Emmanuel Mubangizi

*Uganda*

Ricardo Muñoz

*United States*

David Musyimi Ndetei

*Kenya*

Charles Newton

*United Kingdom*

Patrick Onyango-Mangen

*Uganda*

Ángel A. Otero-Ojeda

*Cuba*

Femi Oyebode

*Nigeria / United Kingdom*

Clare Pain

*Canada*

Vikram Patel

*United Kingdom*

Sergio Pérez-Barrero

*Cuba*

Michael Robert Phillips

*Canada / United States / China*

Jennifer Pinto-Martin

*United States*

Cas Prinsloo

*South Africa*

Dainius Puras

*Lithuania*

Atif Rahman

*Pakistan / United Kingdom*

Shoba Raja

*India*

Brian Robertson

*South Africa*

Graciela Rojas-Castillo

*Chile*

Elizabeth Saewyc

*Canada*

Sandra Saldivia-Bórquez

*Chile / Spain*

Walid Sarhan

*Jordan*

Shekhar Saxena

*Switzerland*

Soraya Seedat

*South Africa*

Rahul Shidhaye

*India*

Siham Sikander

*Pakistan*

Dartiu Xavier da Silveira Filho

*Brazil*

Rune J. Simeonsson

*United States*

Shoba Srinath

*India*

Nirmala Srinivasan

*India*

Ezra S. Susser

*United States*

Manickam Thirunavukarasu

*India*

Wietse Tol

*Netherlands / United States*

Mark Tomlinson

*South Africa*

Sandra Tsang Kit-Man

*China*

Pietro Paolo Turrone

*Canada*

Richard Uwakwa

*Nigeria*

Nadja Van Ginneken

*United Kingdom*

Mark Van Ommeren

*Netherlands / Switzerland*

Horacio Vargas-Murga

*Peru*

Peter Ventevogel

*Netherlands*

Wendy Margaret Vogel

*South Africa*

Lize Weich

*South Africa*

Lisa Wexler

*United States*

Christopher Wilkes

*Canada*

Shuiyuan Xiao

*China*

Marshalyn Yeargin-Allsopp

*United States*

Xudong Zhao

*China*

Yi Zheng

*China*
