# Supplementary material for: The ethical, social, and cultural dimensions of screening for mental health in children and adolescents of the developing world
Source: PLoS One. 2020 Aug 24;15(8):e0237853. doi: 10.1371/journal.pone.0237853 (PMC7446846; doi:10.1371/journal.pone.0237853)
Supplement: S2 Table — (DOCX) [file pone.0237853.s002.docx]

**Supporting Information**

# S2 Table. Number of panelists who completed all rounds

# of the Delphi, by country

| Countries | Number of panelists |
| --- | --- |
| United States | 19 |
| South Africa | 17 |
| Brazil | 11 |
| Canada | 10 |
| India | 9 |
| United Kingdom | 7 |
| China | 4 |
| Nigeria | 4 |
| Uganda | 4 |
| Chile | 3 |
| Cuba | 3 |
| Kenya | 3 |
| Mexico | 3 |
| Netherlands / United Kingdom | 2 |
| Pakistan | 2 |
| Peru | 2 |
| Switzerland | 2 |
| Argentina | 1 |
| Cambodia / Sweden | 1 |
| Canada / United States / China | 1 |
| Ethiopia | 1 |
| France / United States | 1 |
| Germany | 1 |
| Haiti | 1 |
| India / Australia | 1 |
| India / Singapore | 1 |
| India / United Kingdom | 1 |
| Iran | 1 |
| Jamaica / United Kingdom | 1 |
| Jordan | 1 |
| Lithuania | 1 |
| Malaysia | 1 |
| Mexico / United States | 1 |
| Netherlands | 1 |
| Netherlands / Switzerland | 1 |
| Netherlands / United States | 1 |
| Niger | 1 |
| Nigeria / United Kingdom | 1 |
| Pakistan / United Kingdom | 1 |
| Peru / Norway | 1 |
| Saint Vincent and The Grenadines | 1 |
| Singapore | 1 |
| Spain / Chile | 1 |
| Tanzania | 1 |
| Trinidad And Tobago | 1 |
| Turkey | 1 |
| United Kingdom / Switzerland | 1 |
|  |  |
| Total | 135 |
