# Supplementary material for: The ethical, social, and cultural dimensions of screening for mental health in children and adolescents of the developing world
Source: PLoS One. 2020 Aug 24;15(8):e0237853. doi: 10.1371/journal.pone.0237853 (PMC7446846; doi:10.1371/journal.pone.0237853)
Supplement: S3 Table — (DOCX) [file pone.0237853.s003.docx]

**Supporting Information**

# S3 Table. Number and percentage of respondents

# and non-respondents to Delphi Round 1,

# by field of expertise

|  | **Respondents**  **(n=165)** | **Non-respondents**  **(n=325)** |
| --- | --- | --- |
| **Fields of expertise** | ***n* (%)** | ***n* (%)** |
| Experts in MH of vulnerable populations | 105 (63.6%) | 151 (46.5%) |
| Experts in global mental health, public health, epidemiology, or health economics | 85 (51.5%) | 112 (34.5%) |
| Physicians, especially psychiatrists and neuropsychiatrists | 83 (50.3%) | 167 (51.4%) |
| Experts in ethical, social, and cultural issues in MH | 77 (46.7%) | 139 (42.8%) |
| MH administrators | 51 (30.9%) | 130 (40.0%) |
| C&A physicians, especially psychiatrists and neuropsychiatrists | 29 (17.6%) | 52 (16.0%) |
| Psychologists, including neuropsychologists | 23 (13.9%) | 61 (18.8%) |
| Members of NGOs and non-profit and philanthropic organizations | 22 (13.3%) | 30 (9.2%) |
| Representatives of international MH societies and associations | 12 (7.3%) | 26 (8.0%) |
| C&A psychologists, including neuropsychologists | 10 (6.1%) | 3 (0.9%) |
| Editors of MH journals | 9 (5.5%) | 16 (4.9%) |
| Experts in MH policies, laws, and regulations | 8 (4.8%) | 11 (3.4%) |
| Experts in MH screening | 6 (3.6%) | 2 (0.6%) |
| Anthropologists | 6 (3.6%) | 12 (3.7%) |
| Experts in the rights of individuals with disabilities | 6 (3.6%) | 13 (4.0%) |
| MH and community advocates | 5 (3.0%) | 20 (6.2%) |
| Social workers | 3 (1.8%) | 6 (1.8%) |
| Occupational therapists | 3 (1.8%) | 1 (0.3%) |
| MH patients, users, and survivors | 3 (1.8%) | 8 (2.5%) |
| Religious leaders | 3 (1.8%) | 1 (0.3%) |
| Nurses, especially those with expertise on C&A | 2 (1.2%) | 2 (0.6%) |
| Bioethicists | 2 (1.2%) | 8 (2.5%) |
| Experts in the rights of C&A | 2 (1.2%) | 1 (0.3%) |
| Teachers with a background in education for special needs students | 1 (0.6%) | 0 (0.0%) |
| Sociologists | 1 (0.6%) | 5 (1.5%) |
| Experts in MH education | 0 (0.0%) | 3 (0.9%) |
