# Supplementary material for: The ethical, social, and cultural dimensions of screening for mental health in children and adolescents of the developing world
Source: PLoS One. 2020 Aug 24;15(8):e0237853. doi: 10.1371/journal.pone.0237853 (PMC7446846; doi:10.1371/journal.pone.0237853)
Supplement: S1 Fig — (DOCX) [file pone.0237853.s004.docx]

**Supporting Information**

# Figure. Heat maps for Challenges Themes II-VIII*

# and Solutions Themes I-VII.

*** The heat map corresponding to Challenge Theme I appears in the Results section of the manuscript**


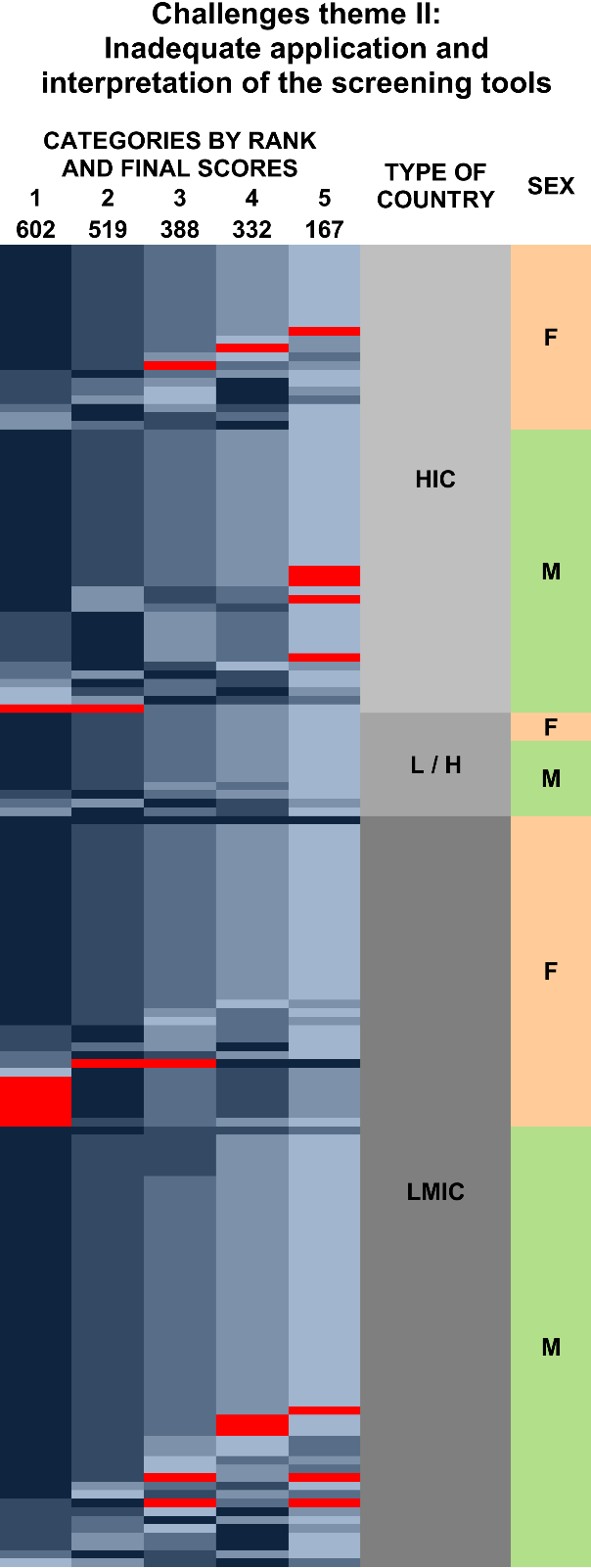


**Challenges theme II: Inadequate application and interpretation of the screening tools**

**Categories by rank**

1. Screening in schools will miss individuals who do not attend educational institutions
2. Screening may be hampered by inaccessibility, cost of transportation, unawareness about the location of the screening, or inconvenient timing
3. Families may wrongly believe that the more screening questions they answer affirmatively, the higher the likelihood of receiving medical, educational, or financial support
4. The screening tools may not be applied or interpreted correctly, particularly if administered by non-specialists or lay people who may make inadequate judgments
5. Adults may fear that intrusive questions about sex or drug use may lead to these behaviours in the individuals screened

|  | **Category ranked first** | **HIC** | **High-income country** |
| --- | --- | --- | --- |
|  | **Category ranked second** | **L / H** | **Mixed** |
|  | **Category ranked third** | **LMIC** | **Low- and middle-income country** |
|  | **Category ranked fourth** |  |  |
|  | **Category ranked fifth** | **F** | **Female** |
|  | **Rejected** | **M** | **Male** |

Figure S4 a. Heat map with the distribution of the final rankings of Challenges theme II: Inadequate application and interpretation of the screening tools identified by the panelists by sex and type of country according to the World Bank classification


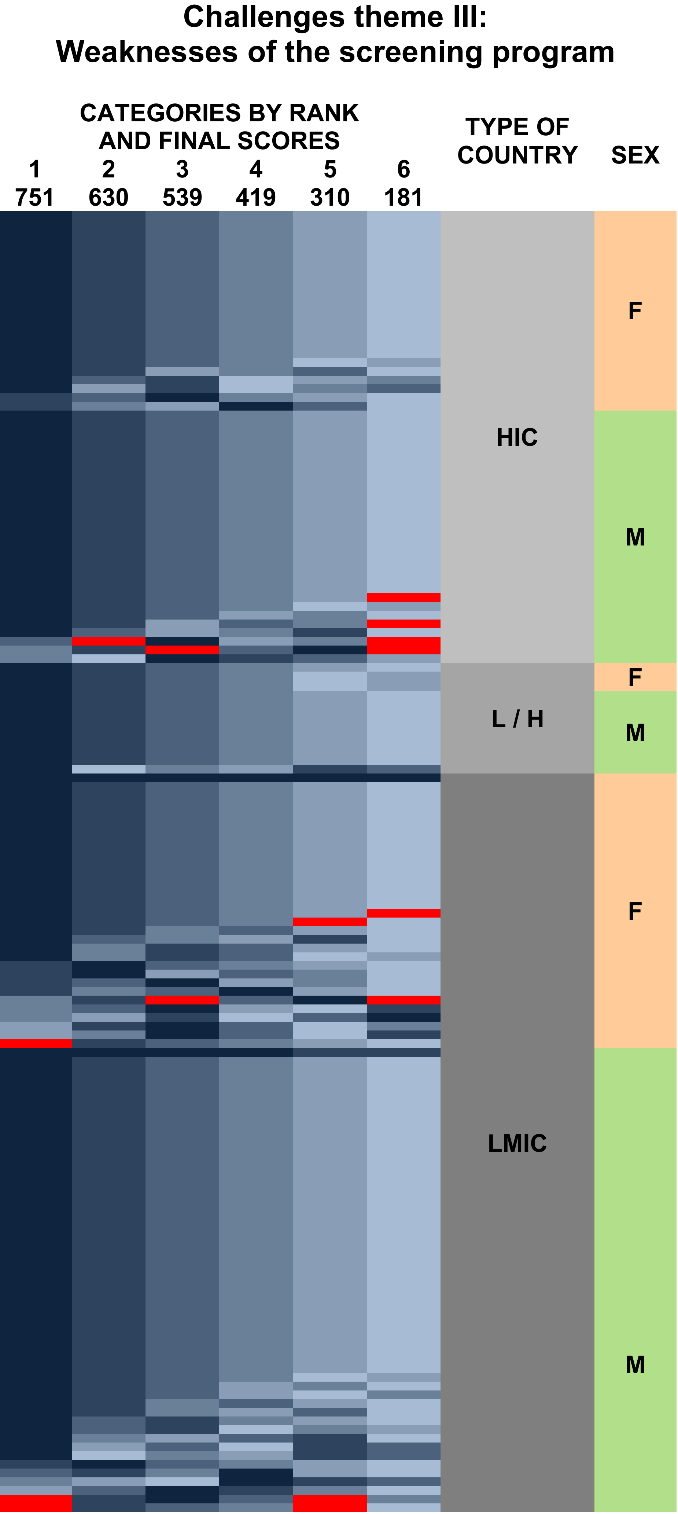


|  | **Category ranked first** | **HIC** | **High-income country** |
| --- | --- | --- | --- |
|  | **Category ranked second** | **L / H** | **Mixed** |
|  | **Category ranked third** | **LMIC** | **Low- and middle-income country** |
|  | **Category ranked fourth** |  |  |
|  | **Category ranked fifth** | **F** | **Female** |
|  | **Category ranked sixth** | **M** | **Male** |
|  | **Rejected** |  |  |

**Challenges theme III: Weaknesses of the screening program**

**Categories by rank**

1. A badly designed, locally-inappropriate screening program may do more harm than good
2. Marginalized populations may not be screened
3. Screening programs may underestimate the contributory causal role of psychosocial determinants of MNSDs such as poverty and all of its various consequences
4. Screening programs may be geared towards the needs of the screeners or researchers, and not of those screened
5. Insufficient evidence exists in LMICs that screening for MNSDs is reliable, cost-effective, or harmless
6. Screening programs may overlook important environmental contributors to MNSDs such as iodide deficiency, tuberculosis, malaria, and HIV / AIDS

Figure S4 b. Heat map with the distribution of the final rankings of Challenges theme III: Weaknesses of the screening program identified by the panelists by sex and type of country according to the World Bank classification


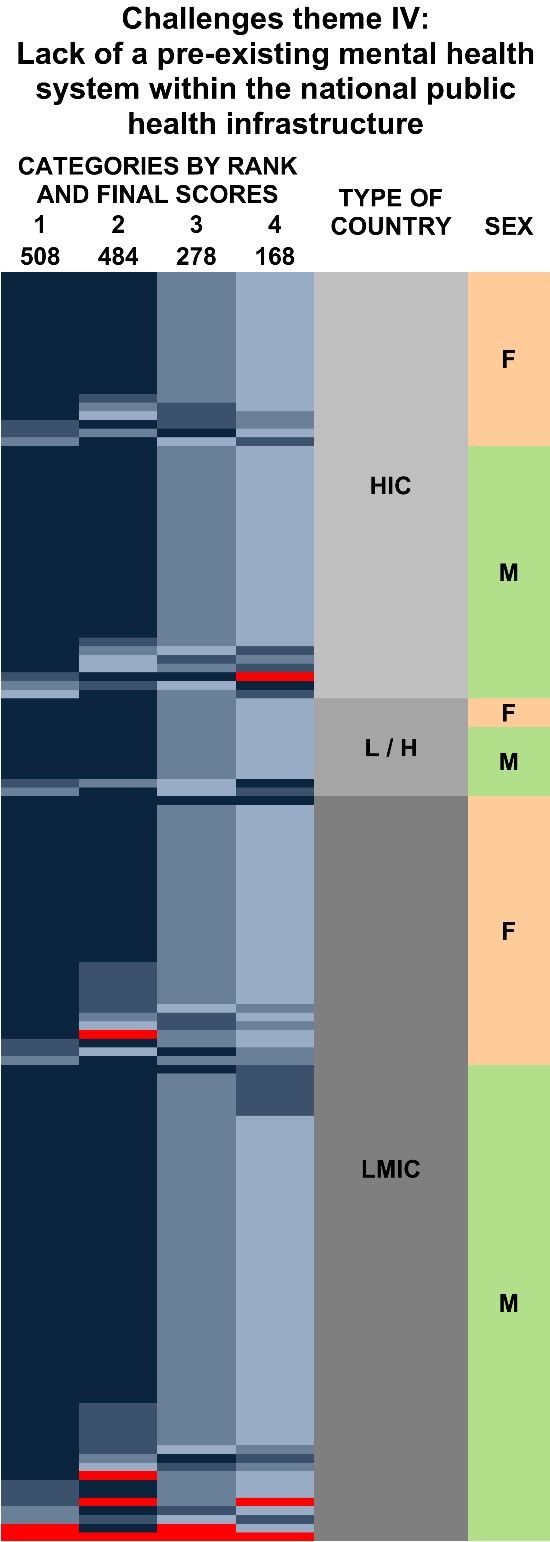


|  | **Category ranked first** | **HIC** | **High-income country** |
| --- | --- | --- | --- |
|  | **Category ranked second** | **L / H** | **Mixed** |
|  | **Category ranked third** | **LMIC** | **Low- and middle-income country** |
|  | **Category ranked fourth** |  |  |
|  | **Rejected** | **F** | **Female** |
|  |  | **M** | **Male** |

**Challenges theme IV: Lack of a pre-existing mental health system within the national public health infrastructure**

**Categories by rank**

1. Screening for MNSDs will not be beneficial if it is not integrated into the primary health care system
2. Screening programs may be hampered by the lack of a national mental health strategic plan that provides adequate funding, administrative and logistic support, and supervision
3. Screening for MNSDs may lack political support and may not be perceived as urgent compared to other priorities by uninformed decision-makers
4. Mental health services in some geographical regions may not be geared towards children and adolescents

Figure S4 c. Heat map with the distribution of the final rankings of Challenges theme IV: Lack of a pre-existing mental health system within the national public health infrastructure identified by the panelists by sex and type of country according to the World Bank classification


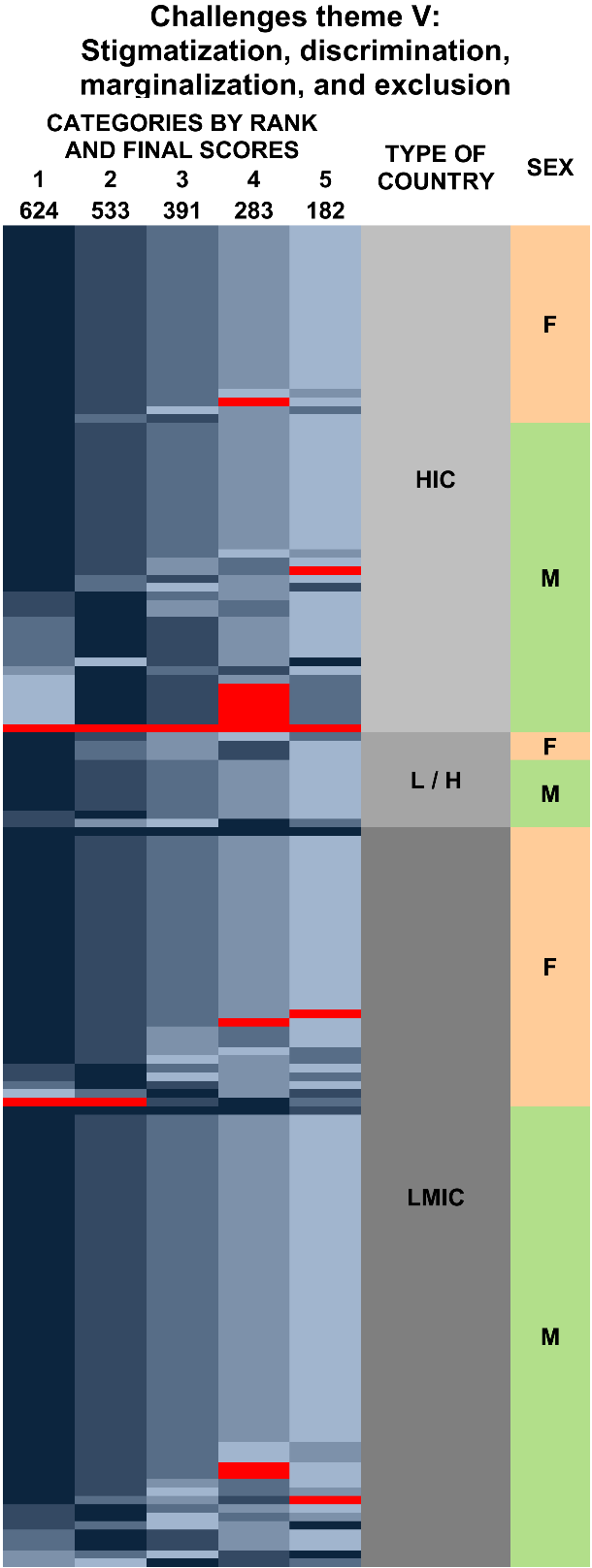


|  | **Category ranked first** | **HIC** | **High-income country** |
| --- | --- | --- | --- |
|  | **Category ranked second** | **L / H** | **Mixed** |
|  | **Category ranked third** | **LMIC** | **Low- and middle-income country** |
|  | **Category ranked fourth** |  |  |
|  | **Category ranked fifth** | **F** | **Female** |
|  | **Rejected** | **M** | **Male** |

**Challenges theme V: Stigmatization, discrimination, marginalization, and exclusion**

**Categories by rank**

1. At-risk individuals, their families, or their communities can be stigmatized, discriminated against, marginalized, or subjected to exclusion and violence
2. Respondents may withhold information out of fear of stigmatization and discrimination for appearing indiscreet or vulnerable (particularly in the case of males)
3. The screening program may be hindered by social, cultural, or religious biases
4. Parents and caregivers of at-risk individuals may be subjected to “blaming and shaming”
5. Individuals who screen positive may suffer exclusion from health services, educational institutions, or jobs

Figure S4 d. Heat map with the distribution of the final rankings of Challenges theme V: Stigmatization, discrimination, marginalization, and exclusion identified by the panelists by sex and type of country according to the World Bank classification

**
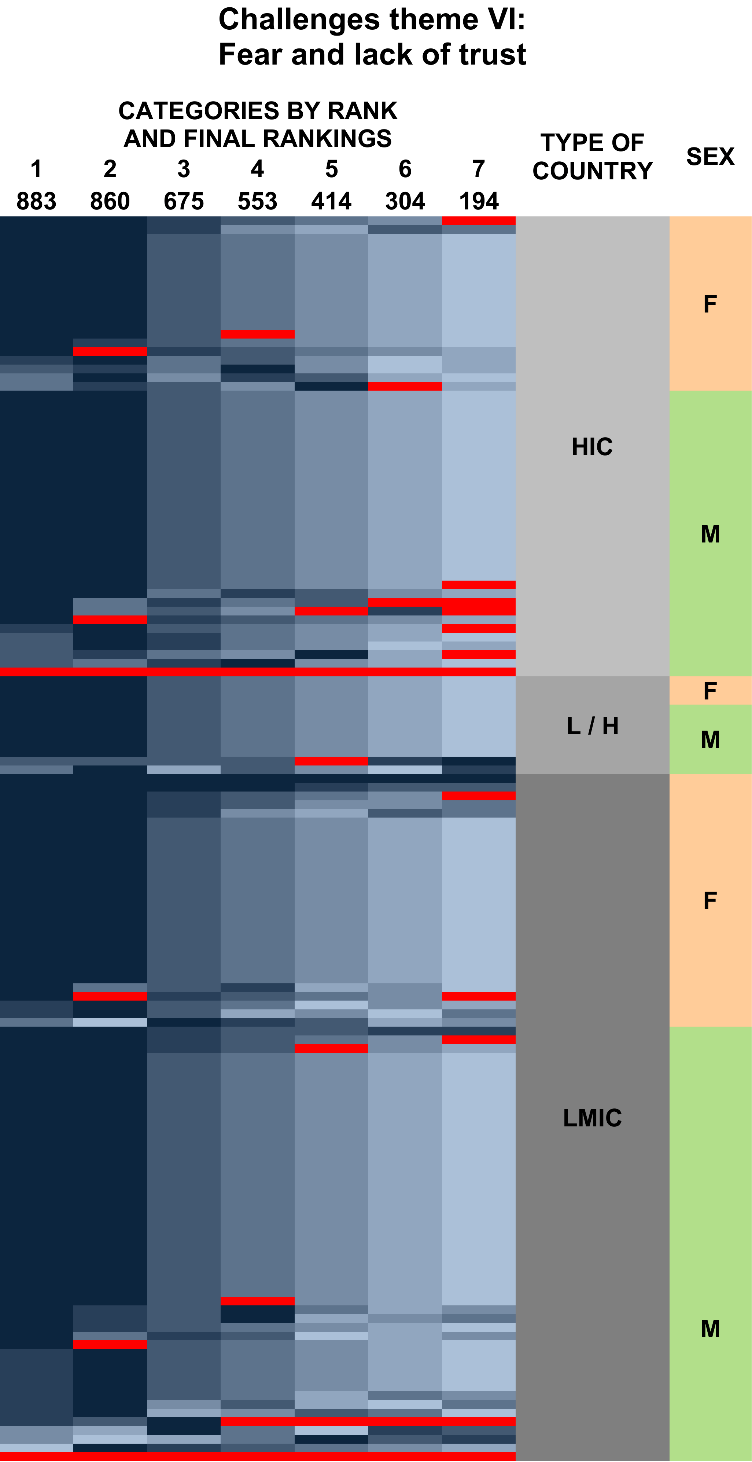
**

|  | **Category ranked first** | **HIC** | **High-income country** |
| --- | --- | --- | --- |
|  | **Category ranked second** | **L / H** | **Mixed** |
|  | **Category ranked third** | **LMIC** | **Low- and middle-income country** |
|  | **Category ranked fourth** |  |  |
|  | **Category ranked fifth** | **F** | **Female** |
|  | **Category ranked sixth** | **M** | **Male** |
|  | **Category ranked seventh** |  |  |
|  | **Rejected** |  |  |

**Challenges theme VI: Fear and lack of trust**

**Categories by rank**

1. Social desirability bias may prevent candid or truthful responses
2. Illiteracy or lack of education may increase suspicion and unwillingness to participate in the screening
3. Screening may be perceived as too intrusive
4. Individuals may not trust screeners, health care workers, or Western medicine in general
5. If screening is performed or organized by foreigners, it may create resentment
6. Parents and caregivers who have themselves suffered from MNSDs and who have been treated cruelly may refuse to have their children screened
7. Parents and caregivers may encourage those screened to under-report MNSD symptoms for fear that their children will be taken away and institutionalized

Figure S4 e. Heat map with the distribution of the final rankings of Challenges theme VI: Fear and lack of trust identified by the panelists by sex and type of country according to the World Bank classification


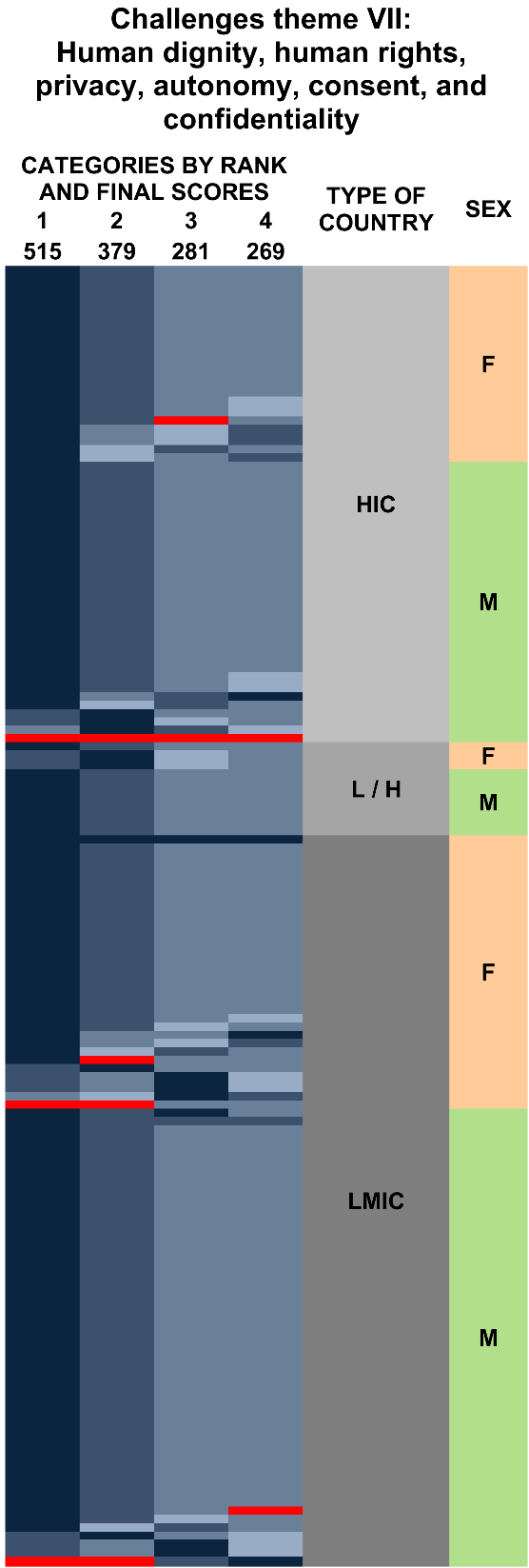


|  | **Category ranked first** | **HIC** | **High-income country** |
| --- | --- | --- | --- |
|  | **Category ranked second** | **L / H** | **Mixed** |
|  | **Category ranked third** | **LMIC** | **Low- and middle-income country** |
|  | **Category ranked fourth** |  |  |
|  | **Rejected** | **F** | **Female** |
|  |  | **M** | **Male** |

**Challenges theme VII: Human dignity, human rights, privacy, autonomy, consent, and confidentiality**

**Categories by rank**

1. Screening programs may not be able to handle the complexities of consent (such as age of autonomy), information sharing with parents and others, and data management (access, storage, protection, and destruction)
2. Free and informed consent of children and adolescents may be ignored
3. Individuals may feel vulnerable in the absence of a guarantee of privacy and confidentiality
4. Genuine informed consent may be compromised by lack of knowledge about MNSDs or by inadequate explanations about the screening process

Figure S4 f. Heat map with the distribution of the final rankings of Challenges theme VII: Human dignity, human rights, privacy, autonomy, consent, and confidentiality identified by the panelists by sex and type of country according to the World Bank classification


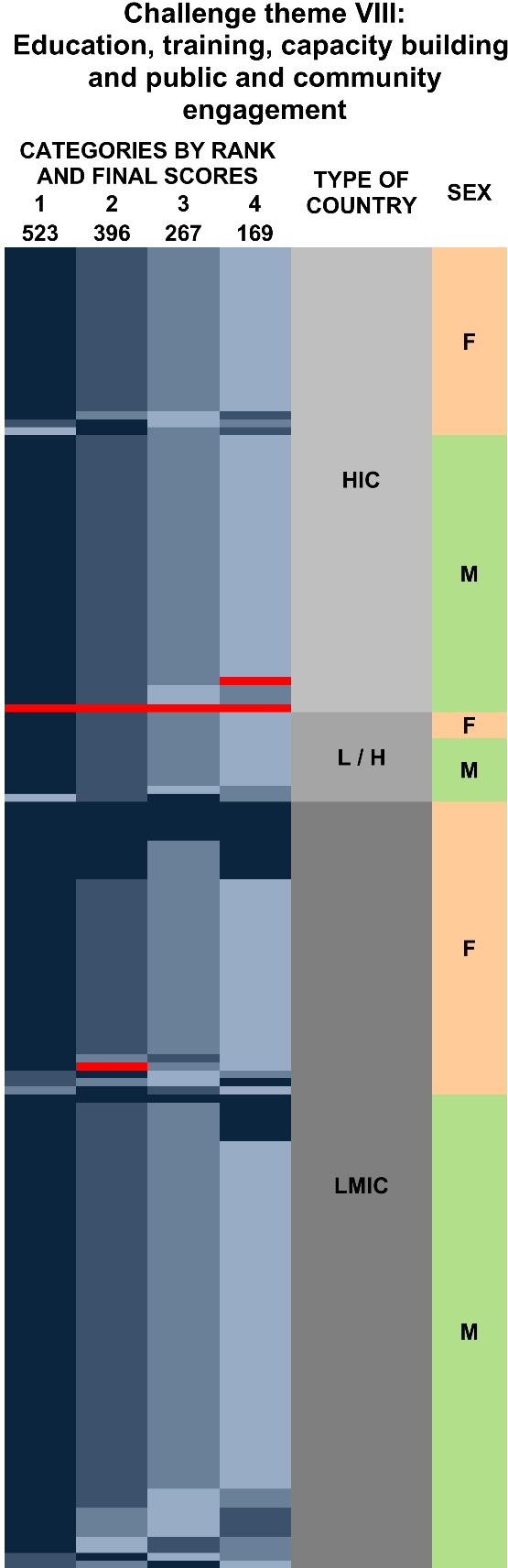


|  | **Category ranked first** | **HIC** | **High-income country** |
| --- | --- | --- | --- |
|  | **Category ranked second** | **L / H** | **Mixed** |
|  | **Category ranked third** | **LMIC** | **Low- and middle-income country** |
|  | **Category ranked fourth** |  |  |
|  | **Rejected** | **F** | **Female** |
|  |  | **M** | **Male** |

**Challenge theme VIII: Education, training, capacity building, and public and community engagement**

**Categories by rank**

1. In most LMICs, there is a severe shortage of specialized mental health care workers and of adequate specialization programs
2. Already overburdened health care workers and teachers may be unmotivated to participate in the screening programs and may resent the encroachment on their everyday duties
3. Caregivers without adequate guidance and support may be unprepared to address the needs of children and adolescents identified as at-risk through the screening
4. In low-resource and low-education settings, properly sensitizing, training, and supervising local non-specialists may be very challenging

Figure S4 g. Heat map with the distribution of the final rankings of Challenge theme VIII: Education, training, capacity building, and public and community engagement identified by the panelists by sex and type of country according to the World Bank classification

**
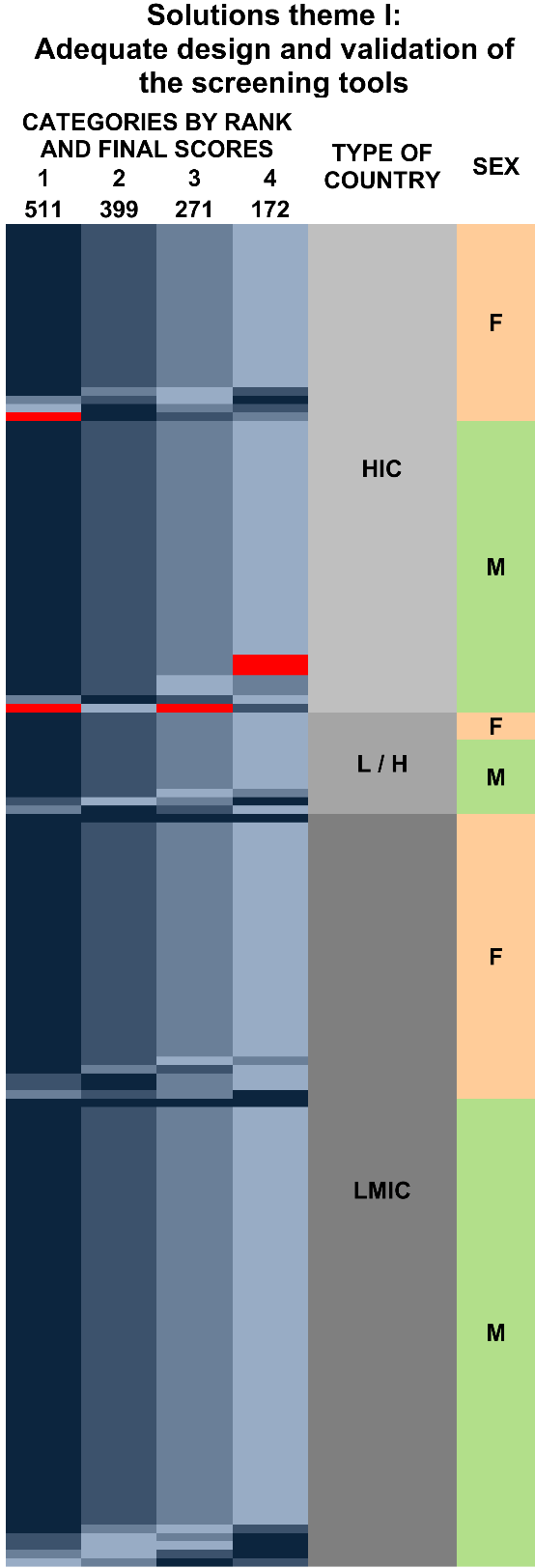
**

|  | **Category ranked first** | **HIC** | **High-income country** |
| --- | --- | --- | --- |
|  | **Category ranked second** | **L / H** | **Mixed** |
|  | **Category ranked third** | **LMIC** | **Low- and middle-income country** |
|  | **Category ranked fourth** |  |  |
|  | **Rejected** | **F** | **Female** |
|  |  | **M** | **Male** |

**Solutions theme I: Adequate design and validation of the screening tools**

**Categories by rank**

1. Develop affordable and scalable screening tools that are easy to understand and simple to administer and that have clear definitions, precise goals, and a well-structured response set
2. Ensure that the screening instruments and rating scales are flexible, evidence-based, culturally and socially valid (focusing on languages and educational levels), and age-appropriate, but standardized to enable cross-cultural comparisons
3. Pilot the screening instruments in relevant local communities
4. Ensure that the screening instruments not only evaluate symptoms but also provide health workers with sufficient information to help at-risk individuals

Figure S4 h. Heat map with the distribution of the final rankings of Solutions theme I: Adequate design and validation of the screening tools identified by the panelists by sex and type of country according to the World Bank classification

**
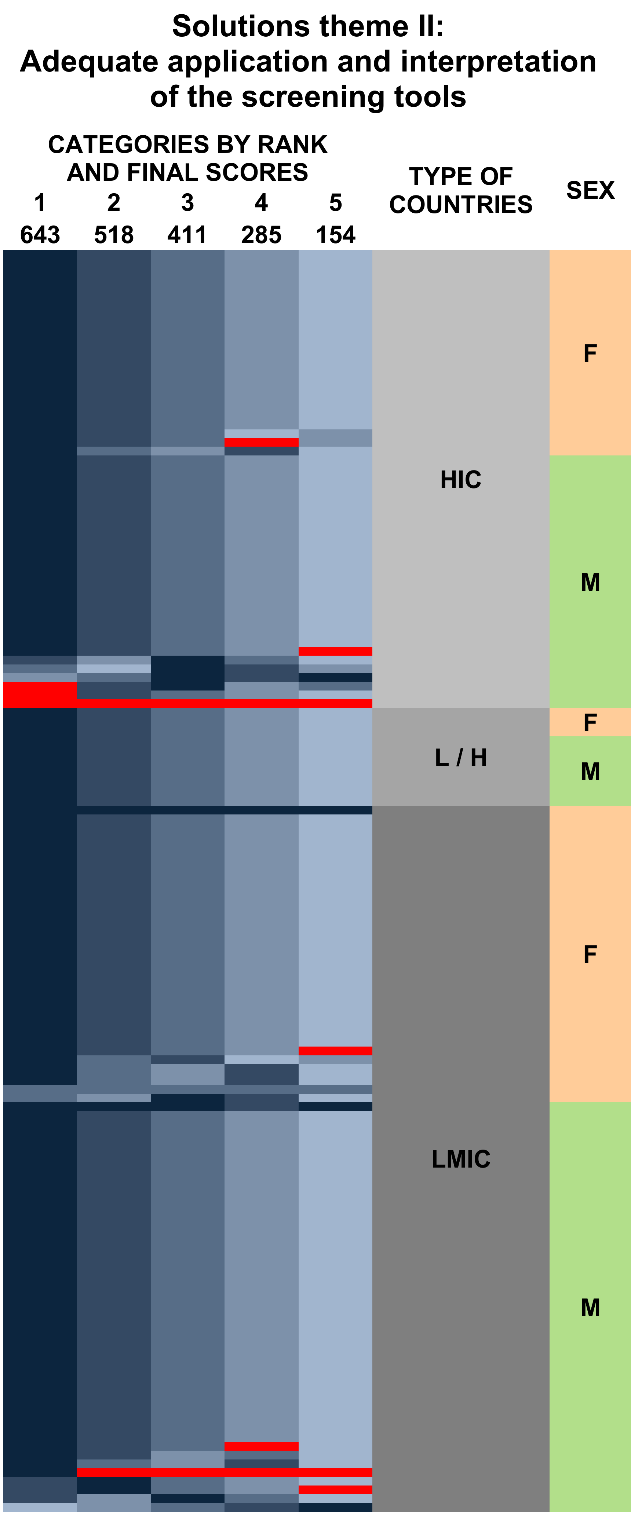
**

|  | **Category ranked first** | **HIC** | **High-income country** |
| --- | --- | --- | --- |
|  | **Category ranked second** | **L / H** | **Mixed** |
|  | **Category ranked third** | **LMIC** | **Low- and middle-income country** |
|  | **Category ranked fourth** |  |  |
|  | **Category ranked fifth** | **F** | **Female** |
|  | **Rejected** | **M** | **Male** |

**Solutions theme II: Adequate application and interpretation of the screening tools**

**Categories by rank**

1. Use qualitative research to determine local views and beliefs on MNSDs such as age-appropriate behaviour and idioms of distress
2. When the instrument needs translation, recruit experienced translators, validate translations through bilingual experts and native speakers of the local language, and use extensive back-translations
3. Choose an accessible, socially and culturally acceptable setting for the screening
4. Use vignette-based descriptions and pictorial explanations to describe difficult concepts
5. Consider using, where appropriate, socially- and culturally-sensitive computer, web-based, and mobile screening tools

Figure S4 i. Heat map with the distribution of the final rankings of Solutions theme II: Adequate application and interpretation of the screening tools identified by the panelists by sex and type of country according to the World Bank classification

**
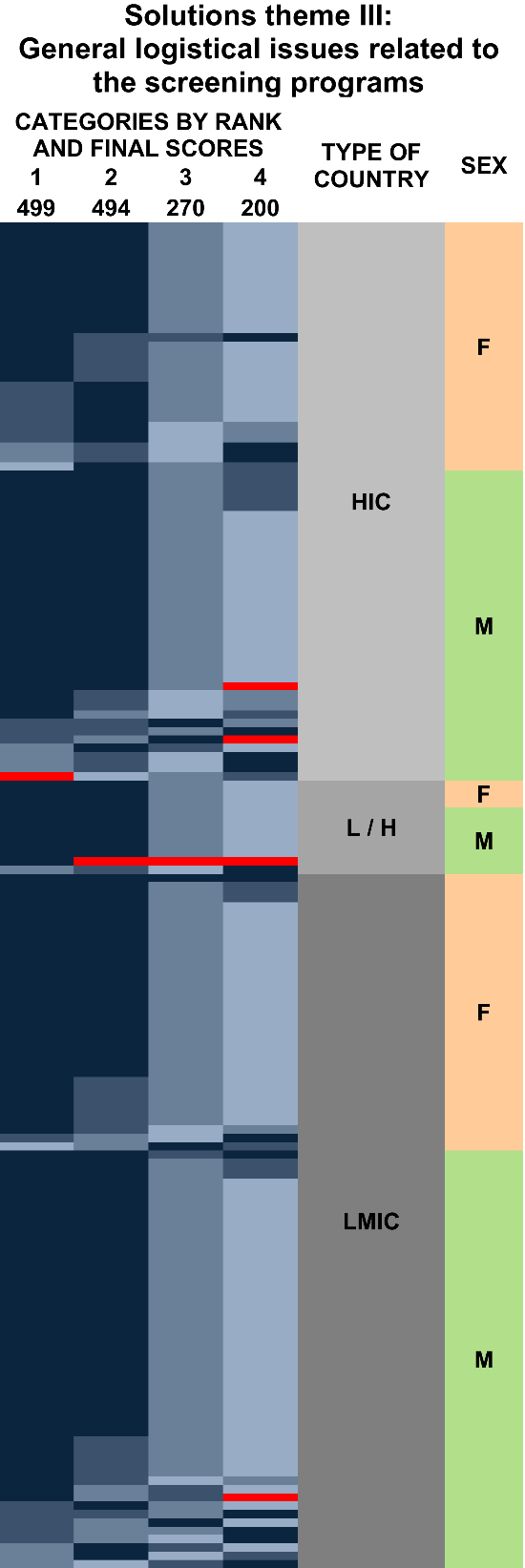
**

|  | **Category ranked first** | **HIC** | **High-income country** |
| --- | --- | --- | --- |
|  | **Category ranked second** | **L / H** | **Mixed** |
|  | **Category ranked third** | **LMIC** | **Low- and middle-income country** |
|  | **Category ranked fourth** |  |  |
|  | **Rejected** | **F** | **Female** |
|  |  | **M** | **Male** |

**Solutions theme III: General logistical issues related to the screening programs**

**Categories by rank**

1. Coordinate the screening programs with general health, education, and social services, and with NGOs and philanthropic institutions
2. Provide adequate explanations to those screened and their families about MNSDs, the screening process, and available therapeutic resources and follow-up, to maximize benefits to at-risk individuals
3. Establish long-term, recurrent screening programs rather than one-off exercises
4. To build trust, incorporate relevant stakeholders at all stages of the screening programs

Figure S4 j. Heat map with the distribution of the final rankings of Solutions theme III: General logistical issues related to the screening programs identified by the panelists by sex and type of country according to the World Bank classification

**
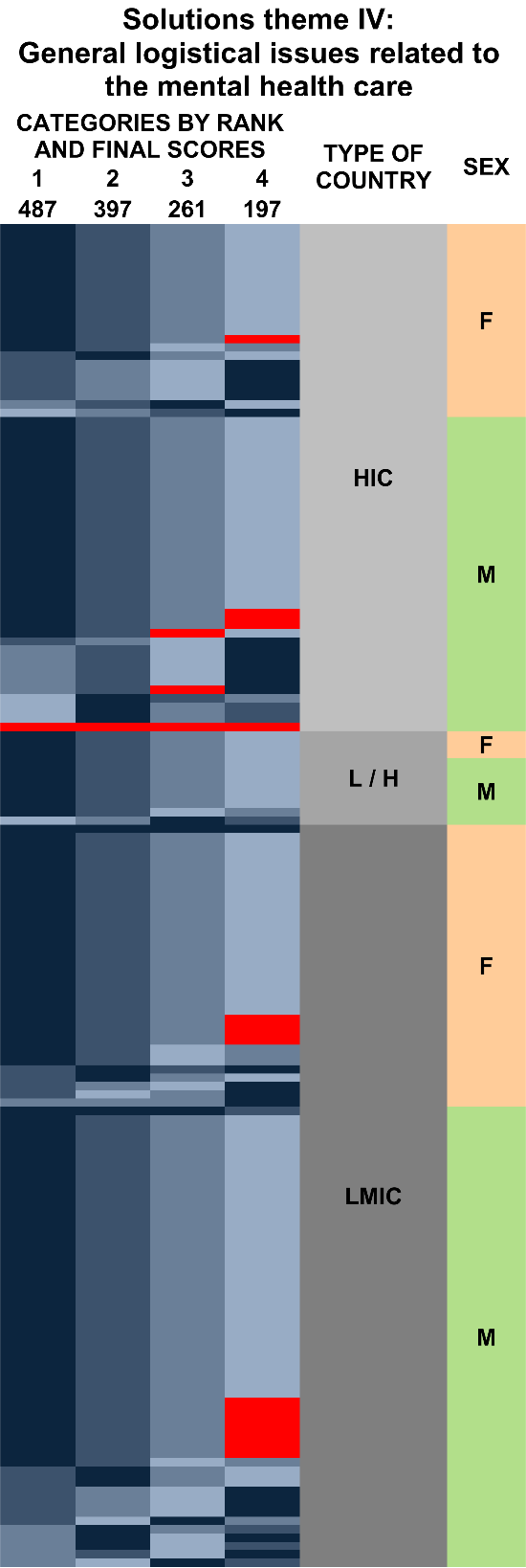
**

|  | **Category ranked first** | **HIC** | **High-income country** |
| --- | --- | --- | --- |
|  | **Category ranked second** | **L / H** | **Mixed** |
|  | **Category ranked third** | **LMIC** | **Low- and middle-income country** |
|  | **Category ranked fourth** |  |  |
|  | **Rejected** | **F** | **Female** |
|  |  | **M** | **Male** |

**Solutions theme IV: General logistical issues related to the mental health care infrastructure**

**Categories by rank**

1. Incorporate screening programs for MNSDs into community-based primary health care and consider integrating them with public health initiatives
2. Ensure that post-screening accessible, affordable, and culturally and socially appropriate treatment and follow-up are available, including early interventions and psychological first aid
3. Facilitate family-, school- and community-based interventions that emphasize resilience and cognitive and emotional skills
4. Only carry out screening for MNSDs if adequate diagnosis, treatment, and follow-up are available

Figure S4 k. Heat map with the distribution of the final rankings of Solutions theme IV: General logistical issues related to the mental health care infrastructure identified by the panelists by sex and type of country according to the World Bank classification

**
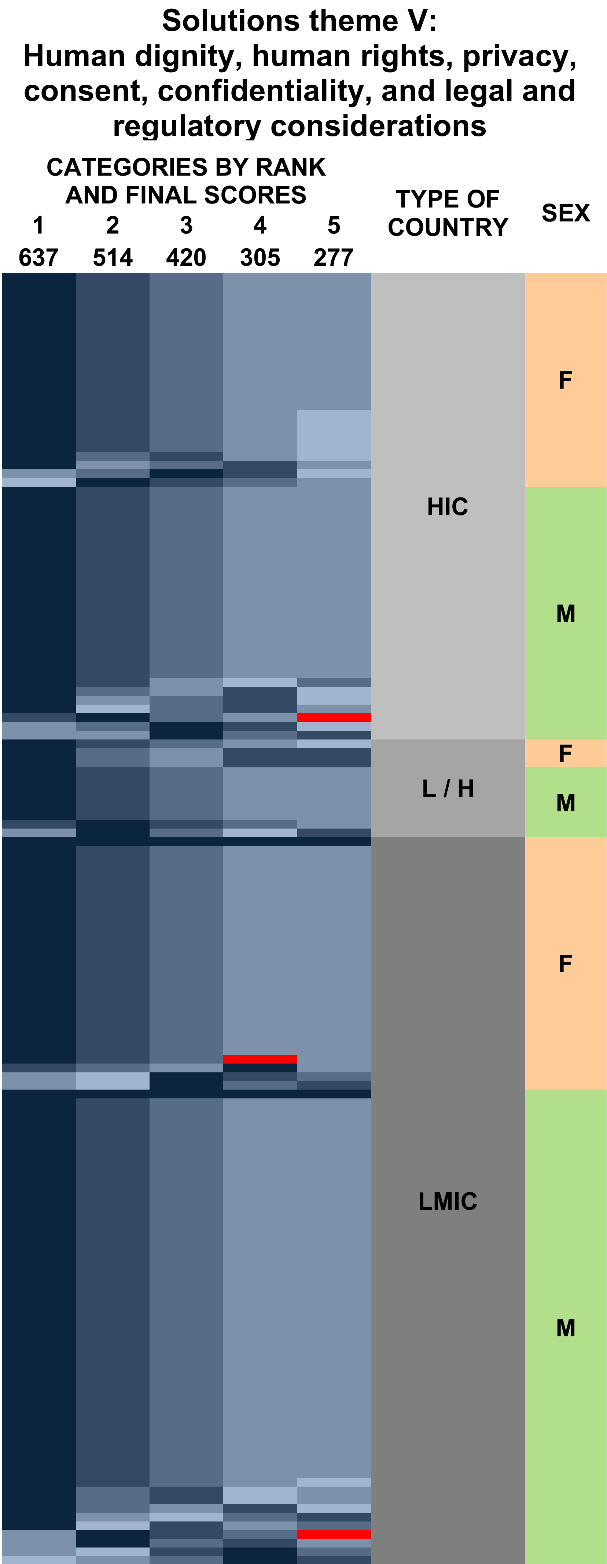
**

|  | **Category ranked first** | **HIC** | **High-income country** |
| --- | --- | --- | --- |
|  | **Category ranked second** | **L / H** | **Mixed** |
|  | **Category ranked third** | **LMIC** | **Low- and middle-income country** |
|  | **Category ranked fourth** |  |  |
|  | **Category ranked fifth** | **F** | **Female** |
|  | **Rejected** | **M** | **Male** |

**Solutions theme V: Human dignity, human rights, privacy, consent, confidentiality, and legal and regulatory considerations**

**Categories by rank**

1. Ensure that screening programs are carried out within a human rights framework that respects the dignity of those screened
2. Anticipate, prevent, and reduce stigmatization, discrimination, marginalization, and neglect of screened and at-risk individuals
3. Guarantee the privacy and confidentiality of all information related to screened individuals and their families, making all concerned aware of this protection
4. Make sure that every screened child and adolescent is regarded as having equality before the law, and that their rights are respected post-screening throughout the mental health system
5. Obtain informed consent or assent for the screening and follow-up of the individual screened without coercion

Figure S4 l. Heat map with the distribution of the final rankings of Solutions theme V: Human dignity, human rights, privacy, consent, confidentiality, and legal and regulatory considerations identified by the panelists by sex and type of country according to the World Bank classification

**
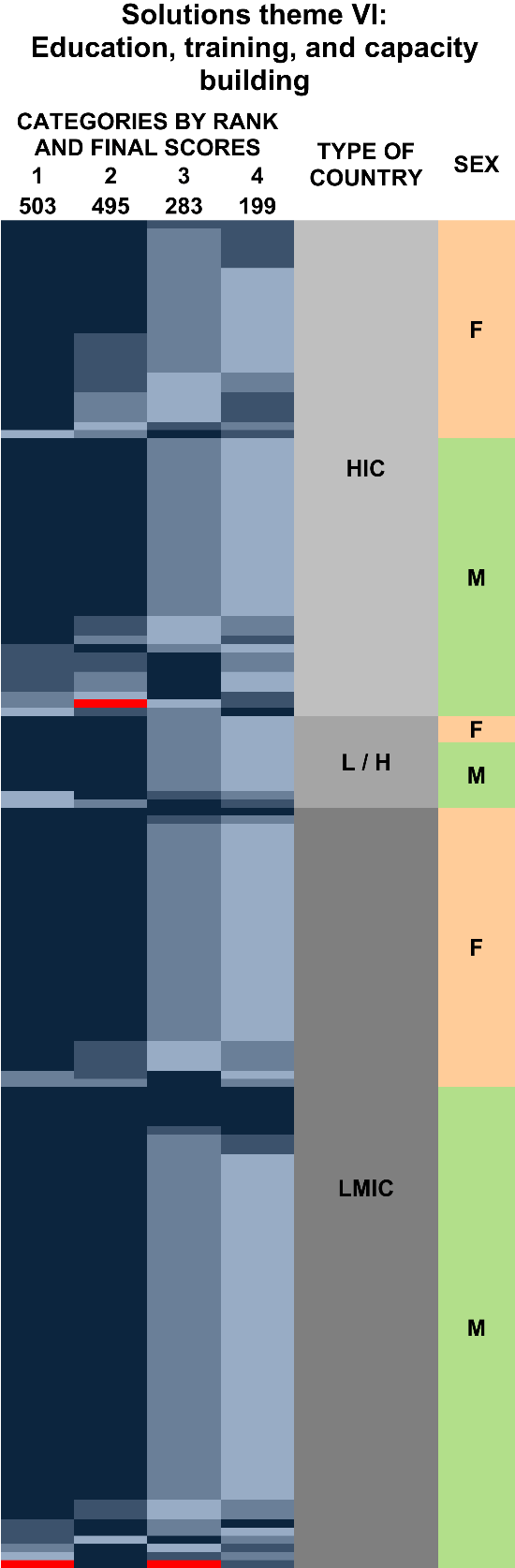
**

|  | **Category ranked first** | **HIC** | **High-income country** |
| --- | --- | --- | --- |
|  | **Category ranked second** | **L / H** | **Mixed** |
|  | **Category ranked third** | **LMIC** | **Low- and middle-income country** |
|  | **Category ranked fourth** |  |  |
|  | **Rejected** | **F** | **Female** |
|  |  | **M** | **Male** |

**Solutions theme VI: Education, training, and capacity building**

**Categories by rank**

1. Provide adequate training, support, and supervision for screeners, making them aware of their own beliefs and biases
2. Train community health workers, especially in remote areas, to carry out screening and affordable and cost-effective primary mental health interventions
3. Include mental health as an integral part of academic curricula at all levels of education
4. Provide adequate training, support, and supervision of all health workers concerned with the care and follow-up of at-risk individuals and their families

Figure S4 m. Heat map with the distribution of the final rankings of Solutions theme VI: Education, training, and capacity building identified by the panelists by sex and type of country according to the World Bank classification

**
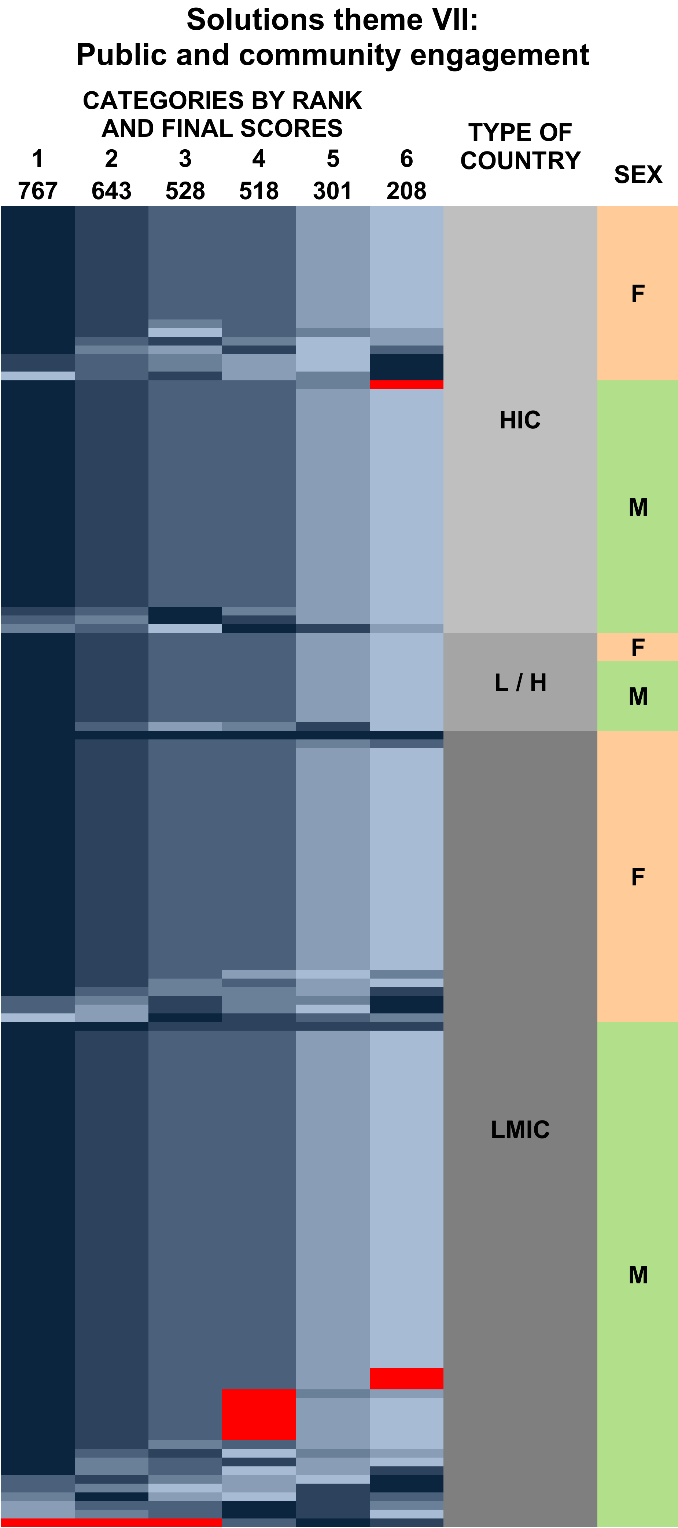
**

|  | **Category ranked first** | **HIC** | **High-income country** |
| --- | --- | --- | --- |
|  | **Category ranked second** | **L / H** | **Mixed** |
|  | **Category ranked third** | **LMIC** | **Low- and middle-income country** |
|  | **Category ranked fourth** |  |  |
|  | **Category ranked fifth** | **F** | **Female** |
|  | **Category ranked sixth** | **M** | **Male** |
|  | **Rejected** |  |  |

**Solutions theme VII: Public and community engagement**

**Categories by rank**

1. Increase the profile of MNSDs by incorporating them into general public health education campaigns
2. Design and carry out at regular intervals culturally-sensitive, adaptable, and empowering public engagement strategies that raise awareness, provide information, address fears, and build trust
3. Engage local communities in respectful dialogue that critically examines the potential benefits and drawbacks of screening programs
4. Identify and engage influential, high-profile local individuals, especially if they have successfully overcome MNSDs, who are willing to participate in public engagement campaigns
5. Consider the use of engagement strategies such as community meetings, home visits, posters, song, dance, and theatre, building upon lessons and experiences from similar previous initiatives
6. Before implementing screening programs for MNSDs, determine the baseline acceptance, motivation, and support for them in target communities

Figure S4 n. Heat map with the distribution of the final rankings of Solutions theme VII: Public and community engagement identified by the panelists by sex and type of country according to the World Bank classification
